# Molecular Mechanisms of Algicidal Bacteria in Controlling Harmful Algal Blooms: Advances in Bacteria‐Algae Interactions

**DOI:** 10.1111/1758-2229.70305

**Published:** 2026-03-02

**Authors:** Jiaxin Wang, Binfu Xu, Lixing Huang

**Affiliations:** ^1^ Fisheries College, Key Laboratory of Healthy Mariculture for the East China Sea, Ministry of Agriculture Jimei University Xiamen Fujian PR China; ^2^ Institute of Biotechnology Fujian Academy of Agricultural Sciences Fuzhou Fujian PR China

**Keywords:** algicidal bacteria, algicidal mechanisms, bacteria‐algae interactions, harmful algal blooms

## Abstract

The frequent occurrence of harmful algal blooms (HAB) poses severe threats to aquatic ecosystems, aquaculture industries and human health. Recently, algicidal bacteria have emerged as a promising biocontrol strategy. However, the precise mechanisms underlying their algicidal effects remain poorly understood, limiting their practical application in environmental management. This review systematically summarises the interactions between bacteria and algae, as well as the various algicidal modes employed by bacteria, with a particular focus on the mechanisms driving bacterial algicidal activity. Key bacterial behaviours such as chemotaxis, adhesion, quorum sensing and the release of extracellular vesicles have been identified as critical factors in the algicidal process, among which the role of bacterial extracellular vesicles warrants special attention. Furthermore, we elaborate on the death mechanisms of algal cells upon bacterial attack, including loss of cellular structural integrity, impairment of photosynthetic systems, oxidative stress responses and disruption of calcium ion homeostasis. Notably, advancements in detection technologies have increasingly highlighted the importance of calcium signalling regulation in algal cell death. This review not only elucidates the molecular mechanisms of bacterial algicidal activity, providing a theoretical foundation for the biocontrol of red tides, but also deepens our understanding of bacteria‐algae interactions.

## Graphical abstract

This review elucidates the molecular mechanisms of algicidal bacteria, highlighting critical behaviours like chemotaxis, quorum sensing and extracellular vesicle release. It details how these actions disrupt algal cellular integrity, photosynthesis and calcium homeostasis, providing a theoretical foundation for controlling harmful algal blooms.

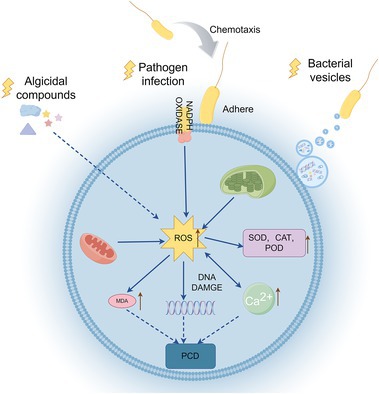

## 1 Introduction

Harmful algal blooms (HABs) refer to phytoplankton outbreaks in aquatic environments, such as marine and freshwater ecosystems, that occur under specific conditions, posing significant threats to the ecological balance of the water. The primary species involved in these blooms include various dinoflagellates (Yu et al. 2023), cyanobacteria (Paerl et al. 2001), diatoms (Smayda 1997), etc. These harmful algae are widely distributed and have been reported across global marine regions, including China, Japan, South Korea, eastern Russia (Sakamoto et al. 2021), the coasts of Northern Europe (Karlson et al. 2021), the United States (Anderson et al. 2021) and the Mediterranean Sea (Zingone et al. 2021). HABs pose significant threats to global ecosystems, aquaculture industries and human health. The extensive proliferation of harmful algae can lead to the depletion of oxygen and other essential nutrients in aquatic environments, jeopardising the survival of other species and disrupting ecological balance (Li et al. 2017). These blooms also cause irreparable economic damage. For instance, in 2012, a bloom of *Karenia mikimotoi* along the Chinese coast resulted in mass mortality of marine organisms, particularly cultured abalone, leading to economic losses of up to $330 million (Li et al. 2017). Similar events in Japan, South Korea and other countries have incurred losses amounting to billions of dollars (Sakamoto et al. 2021). Beyond the impacts on aquaculture, the toxins produced by these harmful algae accumulate in filter‐feeding shellfish, and consumption of these contaminated organisms by humans can lead to poisoning. In addition to impacting fisheries, toxins produced by these harmful algae can accumulate in marine organisms such as shellfish through feeding. When humans consume contaminated seafood, poisoning may occur, leading to public health concerns. These toxins are commonly categorised into five groups based on their clinical symptoms: ciguatera fish poisoning (CFP), paralytic shellfish poisoning (PSP), neurotoxic shellfish poisoning (NSP), amnesic shellfish poisoning (ASP) and diarrhetic shellfish poisoning (DSP) (Grattan et al. 2016). This is sorted out in a review published by Grattan et al. (2016). For instance, ingestion of fish containing CFP toxins can lead to acute gastrointestinal symptoms within 24 h, potentially accompanied by cardiovascular or neurological complications. The other four toxin types primarily accumulate in shellfish. PSP typically causes oral numbness or tingling shortly after consumption, which may progress to facial numbness, along with headache, abdominal pain, nausea, vomiting, dizziness and paresthesia. NSP induces acute gastroenteritis and neurological symptoms such as paresthesia and vertigo, although most patients recover within a short period. ASP presents with acute symptoms including vomiting, abdominal cramps, diarrhoea and headache, and may also involve memory loss. DSP is mainly characterised by severe diarrhoea accompanied by nausea, vomiting and abdominal cramps (Grattan et al. 2016). Beyond direct consumption of contaminated seafood, toxins from HABs can also become airborne and affect humans via aerosol transmission (Lim et al. 2023). Furthermore, the accumulation of toxins in water bodies compromises the quality of water used for human consumption (Brooks et al. 2016; Qin et al. 2010). Despite increased awareness of environmental protection in recent years, these risks remain poorly controlled. Survey data indicate that the frequency of coastal red tide events in the Northern South China Sea (NSCS) increased from 76 occurrences in 1998–2007 to 90 occurrences in 2008–2018 (Liu et al. 2025). Globally, HABs have increased in frequency since the 1980s (Feng et al. 2024), and projections indicate that the number of days with cyanobacterial blooms in large reservoirs and lakes in the United States could rise from 7 days in 2017 to between 18 and 39 days by 2090 (Chapra et al. 2017). Although improvements in the monitoring and reporting systems for HABs may introduce a positive bias in trend estimates, climate warming (Gobler et al. 2017) and nutrient inputs (Wang et al. 2023; Lan et al. 2024) are still likely to increase the frequency of HAB events. Rising global atmospheric CO_2_ levels and elevated temperatures lead to ocean warming, acidification and deoxygenation (Gobler 2020). Under these changing conditions, certain harmful algal species thrive: cyanobacteria, due to their adaptation to warmer waters, often dominate in freshwater systems (Paerl and Huisman 2008), while CO_2_‐limited harmful dinoflagellates may benefit from elevated CO_2_ concentrations and thus gain a competitive advantage (Reinfelder 2011). Furthermore, there is an observed trend of HAB expansion towards polar regions as sea temperatures rise (Griffith et al. 2019). Nutrient enrichment from agricultural, industrial and urban wastewater discharges promotes eutrophication, enabling rapid algal growth driven by nitrogen and phosphorus availability. This is especially evident in freshwater systems close to urban areas, where cyanobacterial blooms frequently occur (Paerl et al. 2001). When these nutrient‐laden freshwaters flow into coastal zones, they can induce sustained eutrophication in marine environments, potentially leading to more severe ecological and economic impacts (Wurtsbaugh et al. 2019). Beyond these primary factors, hydrodynamic conditions such as poor water circulation, along with extreme weather events, including coastal typhoons, heavy rainfall, droughts or prolonged sunny periods, can also trigger algal bloom formation (Feng et al. 2024; Aoki et al. 2019). In summary, the occurrence of algal blooms results from a complex interplay of environmental and anthropogenic factors.

Current strategies to control HABs are largely physical, including ultrasound treatment (Kong et al. 2022), photocatalysts (Wei et al. 2024), and UV‐C irradiation (Gallardo‐Rodríguez et al. 2019). Chemical approaches rely on materials such as clays to promote flocculation and sedimentation of blooms (Jiang et al. 2021), while other chemical methods employ plant extracts (Ni et al. 2023) and aquatic environmental remediation agents (Chai et al. 2025; Zhou et al. 2014) to treat affected waters. Biocontrol options are also being explored, including the use of algicidal bacteria (Yu et al. 2024), algal parasites (Li, Song, et al. 2014), algicidal viruses (Takano et al. 2018) and protists (Li, Gu, et al. 2024). However, physical and chemical methods exhibit several drawbacks: slower action, variable efficacy, higher costs, limited scope, greater energy demands, potential environmental pollution and limited specificity (Balaji‐Prasath 2022; Anabtawi et al. 2024). In contrast, biocontrol approaches, particularly those employing algicidal bacteria, offer environmental compatibility and favourable cost‐effectiveness and are regarded as a promising avenue for HAB mitigation (Anabtawi et al. 2024). Currently, algicidal bacteria can be immobilised for the removal of algal blooms in natural environments. For example, one study immobilised the algicidal bacterium *Shewanella* sp. IRI‐160 on a porous substrate, which preserved its algicidal activity while allowing for recovery after use, thereby minimising environmental impact (Wang and Coyne 2020). Although progress has been made in using algicidal bacteria for algal bloom control, the complexity of natural ecosystems often leads to discrepancies between laboratory results and field applications (Noh et al. 2017). Addressing these challenges will be crucial for advancing real‐world implementation in future applied research.

The relationship between bacteria and microalgae has long been a focal point in environmental research (Azam and Malfatti 2007; Buchan et al. 2014; Hu et al. 2025). As primary producers in marine ecosystems, microalgae utilise photosynthesis to fix inorganic carbon (Field et al. 1998) and release substantial amounts of organic matter into their surroundings. These extracellular metabolites not only attract bacteria to colonise the phycosphere, the zone immediately surrounding algal cells, but also shape bacterial community composition (Patidar 2025). Bacteria and microalgae engage in intricate interactions that significantly influence algal metabolism and growth dynamics. Certain bacteria establish interdependent relationships with algal cells, some of which can reside within the cell envelope or periplasmic space (Coale et al. 2024). Others communicate via chemical signalling or exchange of metabolites (Amin et al. 2015). Additionally, bacteria can release bioactive compounds that inhibit algal growth (Li, Zhu, et al. 2014) or even induce algal cell lysis (Zeng et al. 2021). Following algal bloom senescence, heterotrophic bacteria play a crucial role in decomposing algal‐derived organic matter (detritus), thereby profoundly shaping pelagic energy fluxes and biogeochemical nutrient cycling (Azam and Malfatti 2007).

To address HABs more effectively, research attention has increasingly turned to algicidal bacteria, microorganisms capable of selectively killing certain algal species. In recent years, numerous algicidal strains have been isolated from diverse aquatic environments, and a range of algicidal compounds have been identified, demonstrating promising potential for bloom mitigation (Zhang et al. 2024; Ding et al. 2021). However, the practical application of these bacteria in natural settings remains constrained by an incomplete understanding of their molecular mechanisms of action. This review therefore focuses on elucidating the molecular basis of bacterial algicidal activity. We systematically examine bacterial‐algal interactions, categorise modes of algicidal action, summarise known molecular mechanisms and describe algal cell death pathways. By synthesising current knowledge in these areas, this work aims to deepen the understanding of bacterial‐mediated algal lysis, clarify the ecological roles of bacteria in algal population dynamics, and provide a foundation for developing more effective and targeted bacterial strategies for HAB control.

## 2 Bacterial‐Algal Interactions

In aquatic environments, particularly in marine ecosystems, the relationship between bacteria and microalgae is both complex and intricate. The concept of the phycosphere is similar to the rhizosphere of plants. The environment surrounding algal cells undergoes modifications due to their metabolic activities. For example, algal respiration can influence the surrounding oxygen levels and pH, while the exudation of organic substances from algae contributes to an organic‐rich environment surrounding the algal cells. This creates an environment with specific spatial dimensions and stability (Seymour et al. 2017). Similar to the rhizosphere of plants, the phycosphere also harbours a diverse assemblage of bacterial taxa. Broadly, the bacterial‐algal relationship can be classified into three categories.

### 2.1 Mutualistic Symbiosis

Soluble organic compounds released into the phycosphere, such as amino acids, carbohydrates, sugar alcohols and organic acids (Seymour et al. 2017), are generally available for bacterial utilisation. Upon entering this microenvironment, bacteria benefit by acquiring nutrients while also positively influencing algal growth, thus fostering a mutualistic relationship. For instance, when nutrient levels are low in the environment, phytoplankton growth heavily depends on nitrogen and phosphorus supplied by bacteria (Cole 1982). Additionally, bacteria can act as providers of nutrients through remineralization, which enhances the bioavailability of trace elements to algae (Amin et al. 2009). Furthermore, the synthesis of essential vitamins, such as vitamin B_12_, required by algae, often relies on the contribution of symbiotic bacteria (Croft et al. 2005). Recent studies indicate that bacteria also influence the dormancy and proliferation of algae. In particular, recent work reports that bacteria, via chemical signalling, can induce vesicle formation in ageing diatoms *Coscinodiscus radiatus* , facilitating the extrusion of reactive oxygen species (ROS), oxidised fatty acids and other deleterious metabolic byproducts from aged cells and thereby enabling renewed proliferation (Deng et al. 2024). Symbiotic bacteria can also play a role in interspecies competition among algae. For example, the addition of the symbiotic bacterium *Bacillus* sp. increases the abundance of *Cyclotella atomus* , a species of the centriae diatom, thereby displacing the previously dominant *Fistulifera pelliculosa* and becoming the new dominant species in the environment (Zhou et al. 2025).

### 2.2 Competition

The nutrients in the algal habitat not only attract symbiotic partners desired by algal cells but also lure opportunistic enemies. The availability of nutrients in the environment is a significant factor influencing the structure of microbial communities and can regulate algal populations (Jia, Lu, et al. 2023). Certain bacteria can suppress algal growth by modulating nutrient availability. For example, in interactions between *Stenotrophomonas maltophilia* , a freshwater oligotrophic bacterium and algal cells, the bacteria produce a hydroxamate‐type siderophore, an amino acid derivative, which limits the uptake of iron by cyanobacteria, thereby negatively impacting their growth (Z. Z. Liu 2014). Of course, algal cells are not easily eliminated; HABs and bloom‐forming algae, in particular, are adept at producing allelopathic compounds to inhibit competitors or outcompete other species for essential nutrients (Coyne et al. 2022). Nitrogen, for instance, is a crucial macronutrient in diatom‐bacteria competition. Benthic diatoms, with their high affinity for ammonia, limit the growth and metabolic activity of ammonia‐oxidising bacteria through competitive exclusion (Martens‐Habbena et al. 2009; Risgaard‐Petersen et al. 2004).

### 2.3 Antagonistic Interactions

Competition between algae and bacteria is not always subtle; in many cases, it escalates into active antagonism. Of particular threat to algal cells are algicidal bacteria, which, upon chemotactic attraction to the phycosphere, can eliminate algal cells to access released nutrients for their own proliferation. These attacks are mediated either through the secretion of algicidal compounds or via direct physical interactions. However, algae are not passive victims; they have evolved multiple strategies to counteract bacterial aggression. Studies have shown that, upon bacterial induction, algae can release a suite of antimicrobial compounds. For instance, diatoms secrete proteins (Paul and Pohnert 2011) and oxidised fatty acids (Ianora et al. 2011) with potent bactericidal properties. Additionally, algae may evade contact with lytic bacteria through dormancy strategies, such as the formation of cysts (Roth et al. 2008). Recent evidence also suggests a novel defence mechanism whereby diatoms reduce their cell size during reproduction to decrease surface area for bacterial adhesion (Cai et al. 2023). Algal cells are also capable of disrupting bacterial quorum sensing (QS) systems. *Chlamydomonas reinhardtii* , for example, produces chromophores that mimic bacterial acyl‐homoserine lactones (AHLs) (Teplitski et al. 2004), and further secretes bromocyanin, a secondary metabolite that catalyses the cleavage of AHL peptide bonds (Vanelslander et al. 2012), thereby interfering with bacterial QS signalling and mitigating virulence. Likewise, certain microalgae in aquatic systems secrete auxin analogues with indole structures that inhibit QS in *Vibrio harveyi* , impairing bacterial motility and biofilm formation (Yang et al. 2017).

With the variations in physicochemical conditions and the physiological state of algal cells, the interaction between bacteria and algae becomes increasingly complex (Zhang et al. 2021). For instance, members of the *Rhodobacteraceae* family, such as *Phaeobacter gallaeciensis* , can establish a potentially mutualistic relationship with the marine coccolithophore *Emiliania huxleyi* through the production of growth‐promoting hormones like phenylacetic acid and antibiotics such as protocatechuic acid. However, when *E. huxleyi* cells undergo senescence, *P. gallaeciensis* switches its role to become an opportunistic pathogen. This transition occurs upon detection of byproducts released during algal cell ageing, such as coumaric acid, prompting the bacterium to release lytic molecules, namely roseobactin A and B, which contribute to algal cell lysis (Seyedsayamdost et al. 2011). Furthermore, rising temperatures can lead to the transformation of symbiotic bacteria into antagonistic ones (Lin et al. 2024). For instance, the bacterium *Tamlana* sp. MS1 enhances the growth of the diatom *Skeletonema costatum* at 20°C, but as the water temperature increases, its algicidal activity intensifies, with a lysis rate reaching up to 92.4% at 25°C. This increased activity is attributed to the enhanced motility and adhesion of the bacteria at higher temperatures, facilitating direct contact with the algal cells and thereby enhancing its algicidal effect. Additionally, literature indicates that the production of algicides by bacteria is influenced by environmental nutrient conditions. Under oligotrophic conditions, such as nitrogen and phosphorus limitation, the production of algicides is promoted, whereas under optimal bacterial growth conditions, the production of such compounds is not required (Ray and Bagchi 2001).

## 3 Modes of Algicidal Activity

To better elucidate the specific mechanisms underlying bacterial algicidal activity, the modes of action can be categorised into direct contact and indirect algicidal strategies. In the direct contact mode, bacteria must physically interact with algal cells to exert their lethal effects. Experimentally, this is typically demonstrated when both washed bacterial cells and their corresponding culture supernatants exhibit algicidal activity, whereas the filtered supernatant, lacking bacterial cells, fails to inhibit algal growth (Shi et al. 2023). Several studies have provided further validation by co‐culturing algae and bacteria in compartmentalised systems in which dialysis membranes or similar barriers physically separate the two organisms, thereby preventing direct cell–cell contact while allowing exchange of metabolites between the compartments (Yu et al. 2024; Zeng et al. 2021). This approach is considered more rigorous, as it excludes bacteria that do not require physical contact but may produce algicidal compounds only in response to chemical cues released by algal cells. In contrast, the indirect mode does not require physical association between bacteria and algal cells. Instead, bacteria secrete diffusible algicidal compounds into the environment. In laboratory, the culture filtrate of bacteria or the bacterial secretions induced by algal metabolites exhibits algicidal activity, resulting in the death of algal cells (Zhang et al. 2024).

These two mechanisms are not entirely exclusive; on the contrary, they may co‐occur (Dai et al. 2024). For instance, *Paucibacter* sp. DH15 exhibits a strong removal effect on *Microcystis aeruginosa* through both sterile supernatants and bacterial suspensions after washing (Le et al. 2022). Additionally, *Streptomyces* sp. HY can simultaneously attack *Anabaena* through direct interaction, indirect interaction and biosorption mechanisms (Xie et al. 2024). The *Enterobacterium* sp. H6 has been shown to primarily exert indirect algicidal effects, with direct action being secondary, particularly against *M. aeruginosa* (Dai et al. 2024). These mechanisms may also vary depending on the host; for example, *Bacillus cereus* CZBC1 removes *Oscillatoria chlorina* and *Oscillatoria tenuis* through direct contact, while it employs indirect methods against *Oscillatoria planctonica* (Hu et al. 2019).

### 3.1 Direct‐Contact Algicidal Mechanisms

**FIGURE 1:**
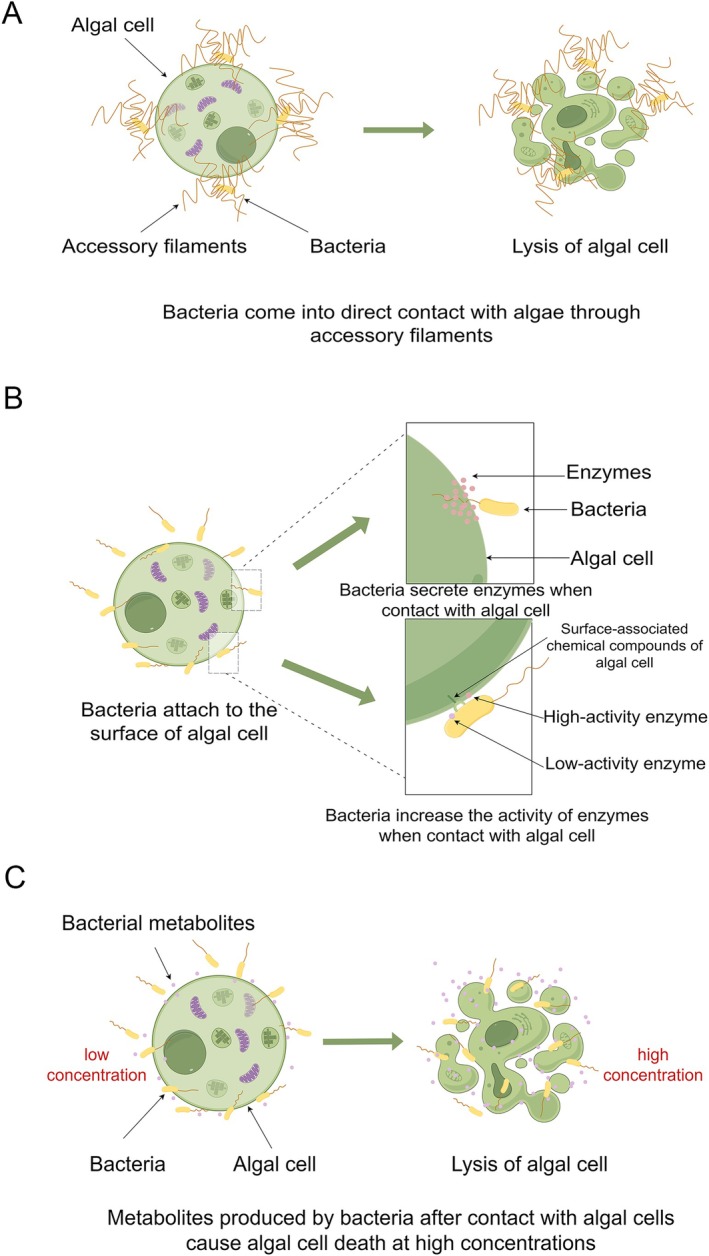
Bacterial mechanisms of algicidal activity through direct contact. (A) *Streptomyces* utilises its hyphal network to target and lyse algal cells. (B) Following contact with algal cells, bacteria secrete proteases or enhance protease activity. (C) After bacterial contact with algal cells, the concentration of algicidal compounds within algal remnants increases.

The known mechanisms of direct contact algicidal activity can generally be classified into three types. The first mechanism involves bacteria with well‐developed appendage‐like filaments, which directly contact and kill algae via these filaments. A classic example is the interaction between the algicidal bacterium *Streptomyces globisporus G9* and the cyanobacterium *M. aeruginosa* , where the bacterium wraps its hyphal filaments around the cyanobacterial cells to capture and kill them. Notably, cyanobacterial cells that are not in direct contact with the hyphae remain morphologically intact (Zeng et al. 2021). Similarly, the filamentous structures of *Saprospira* sp. *SS98‐5*, a saprophytic spirochete bacterium, can capture diatom cells, dissolve the diatom frustule at the point of contact, and subsequently invade and lyse the diatom cells (Furusawa et al. 2003). Literature suggests that this direct invasion mechanism shares similarities with bacterial predation behaviour (Bauer and Forchhammer 2021) (Figure 1A).

The second mechanism occurs when bacteria only generate active algicidal compounds upon contact with molecular structures on the surface of algal cells. For example, *Chitinimonas prasina* LY03 uses its flagella to anchor itself to the surface of algal cells. Upon attachment, it produces chitinase that degrades the algal cell wall (Li, Lei, et al. 2016), Similarly, the free‐living *Alteromonas* sp. *L15* does not exhibit high protease activity unless it adheres to algal cells, at which point it maintains high protease activity capable of hydrolyzing the glycoproteins in the cell wall. This suggests that the chemical cues on the surface of *Thalassiosira pseudonana* cells may play a crucial role in triggering the protease activity of *L15* (Cai et al. 2023) (Figure 1B).

The third mechanism involves bacterial metabolites accumulate around algal cells after contact, increasing local concentrations and potentially reaching algicidal doses. For example, *Roseobacter* sp. can adhere to the green alga *E. huxleyi* , initially utilising tryptophan secreted by the algae to enhance the production of the plant hormone indole‐3‐acetic acid (IAA). At this stage, the concentration of IAA does not harm the algal cells. However, after co‐culturing for 3 weeks, when the concentration of IAA reaches a threshold, it becomes lethal to the algal cells (Segev et al. 2016) (Figure 1C).

### 3.2 Indirect Bacterial Algicidal Mechanisms

**FIGURE 2:**
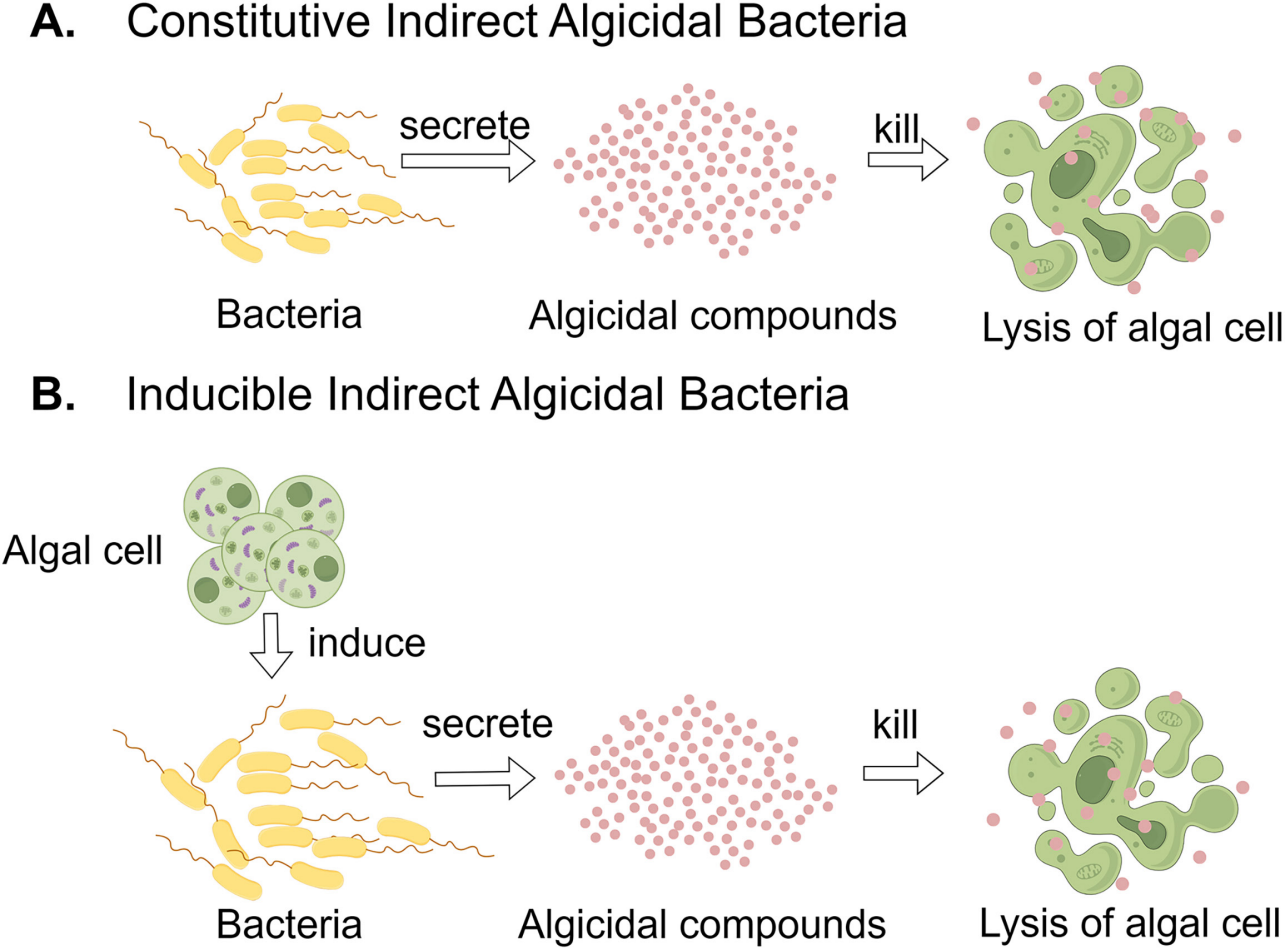
Constitutive and inducible indirect algal toxic bacteria.

Indirect algicidal activity refers to bacterial inhibition or killing of algal cells without physical contact, instead relying on the secretion of algicides. These secreted compounds may be constitutive (Whalen et al. 2018), metabolites inherently toxic to algal cells, or inducible, in which case the production of algicidal substances is triggered by specific algal‐derived molecules. For example, the algal metabolite dimethylsulfoniopropionate (DMSP) can induce bacteria to produce algicides, thereby enabling bacteria to acquire additional sulphur resources (Wang and Seyedsayamdost 2017; José 2018) (Figure 2).

To date, a wide diversity of indirect algicides has been identified. Broadly, these include alkaloids, amino acid derivatives, peptides and proteins, enzymes, polyketides, terpenoids, fatty acids and their derivatives, etc. (Li, Xing, et al. 2024). Yang et al. (2025) compiled a comprehensive survey of approximately 250 natural algicidal compounds isolated from bacteria between the 1960s and 2024, categorising them into algal‐associated and incidental agents, and further distinguishing their relative algicidal potency.

These algicides act on diverse cellular targets. For instance, 79 derivatives were synthesised from the parent compound *N* ^1^‐benzyl‐*N* ^3^, *N* ^3^‐diethylpropane‐1,3‐diamine to probe structure–activity relationships and infer specific sites of action (Park et al. 2023). Another example is the γ‐proteobacterium *Microbulbifer* sp. RZ01, which produces 3,3′,5,5′‐tetrabromo‐2,2′‐dihydroxydiphenyl (4‐BP). This compound exerts algicidal effects by inhibiting plastoquinone biosynthesis, thereby disrupting multiple essential metabolic pathways in phytoplankton (Zhang et al. 2023). Genes responsible for 4‐BP biosynthesis are widespread among diverse bacterial taxa in marine environments, suggesting that it may represent a globally relevant bacterial tool for mediating antagonistic bacteria–algae interactions. Mechanistic studies have revealed that 4‐BP competes with homogentisate for the active site of homogentisate solanesyltransferase (HST), a key enzyme in the plastoquinone biosynthetic pathway, ultimately suppressing photosynthesis and impairing algal viability.

## 4 Molecular Mechanisms Underlying Bacterial‐Mediated Algal Lysis

In aquatic environments, bacteria associated with algal cells do not kill algae by a simple secretion of algicidal compounds. Instead, they need to sense and locate planktonic algae, mobilise towards them and even enter the phycosphere; upon contacting algal cells, they actively or passively regulate their own behaviour to release algicidal substances or to enact other algicidal actions. This process constitutes an integrated, system‐level programme of resource acquisition and interaction with the algae (Meyer et al. 2017). In this section, we summarise the molecular mechanisms that underlie bacterial behaviour during algicidal activity, including how bacteria sense cues from algae, approach and adhere to algal cells, how collective (group) behaviours contribute to algicidal function, and the emerging mechanism of vesicle‐mediated delivery of algicidal substances.

### 4.1 Bacterial Chemotaxis Towards Algal Cells

Chemotaxis refers to the ability of bacteria to sense environmental chemoattractants or chemorepellents and modulate flagellar rotation to direct their movement accordingly, thereby adapting to environmental cues. In the context of bacterial algicidal activity, chemotaxis primarily serves to reduce the physical distance between bacteria and algal cells, enhancing the efficiency of attack (Xu, Ali, et al. 2024). Similar to the rhizosphere bacteria that chemotactically respond to plant‐derived signals, algal cells and their extracellular products act as attractants for algicidal bacteria (Seymour et al. 2017, 2009). These extracellular products are mainly low‐molecular‐weight compounds such as amino acids, monosaccharides, organic acids and secondary metabolites (Bell and Mitchell 1972; Sjoblad and Mitchell 1979). Recent studies have identified oxygen as an additional chemoattractant for bacteria targeting algae (Prakash et al. 2025). Among these chemoattractants, DMSP has been reported as a potent attractant for multiple marine algicidal bacteria (Seymour et al. 2010). The DSYE gene responsible for DMSP synthesis is present in a wide range of algal taxa (Wang et al. 2024), suggesting that DMSP is a broadly conserved chemical cue mediating bacteria–algae interactions in marine environments. Furthermore, the addition of DMSP in bacteria–algae co‐culture systems can enhance bacterial algicidal potency (José 2018), implying a potential role in bloom regulation (Xu, Ali, et al. 2024). Beyond such common attractants, different bacterial species display distinct preferences when interacting with dinoflagellates (Yang et al. 2024). For instance, *Polaribacter marinivivus* and *Lentibacter algarum* preferentially assimilate amino acids and dipeptides, whereas *Litoricola marina* favours nucleotides (Han et al. 2021). Algal cells can also release macromolecules such as glycoproteins, although the secretion of these larger compounds generally lags behind that of small molecules and is more prominent during bloom decline (Seymour et al. 2017). Although less studied, chemotaxis towards algal macromolecules has also been documented. Marine bacteria exhibit strong chemotactic responses to kelp‐derived polysaccharides, providing direct evidence that bacteria can actively target abundant polymeric substrates. Highly chemotactic strains often belong to *Pseudomonadaceae*, *Alteromonadaceae* and *Pseudoalteromonadaceae*, taxa with notable algicidal capabilities. It has been hypothesised that kelp polysaccharides may strongly attract algicidal bacteria during bloom senescence. Intriguingly, pre‐exposure to DMSP can enhance bacterial chemotaxis towards kelp polysaccharides, likely by providing sufficient methyl donors to increase the sensitivity of methyl‐accepting chemotaxis proteins (MCPs), thereby amplifying chemotactic responsiveness (Clerc et al. 2023). In addition to secreted products, algal cells themselves possess inherent attractant properties. For example, in the chemotaxis of *Vibrio coralliilyticus* towards *K. mikimotoi*, the intact algal cells were found to strongly attract bacteria, with membrane components identified as key mediators of this effect (Yu et al. 2024).

Unlike stationary plant cells, planktonic algae in aquatic environments are not merely fixed in place. They are passively displaced by turbulence (Cencini et al. 2019), and some planktonic algae possess flagella and exhibit motility (Basterretxea et al. 2020). This dynamic behaviour places higher demands on the motility capabilities of associated bacteria, which must relocate and navigate to encounter and interact with their algal hosts. Marine bacteria often exhibit chemotactic responses more than an order of magnitude faster than the model organism *Escherichia coli* (Stocker et al. 2008), with greater swimming speeds and directional persistence (Stocker et al. 2008; Stocker and Seymour 2012; Garren et al. 2014; Son et al. 2016). This enables them to closely track motile algal cells (Barbara and Mitchell 2003). Certain species, such as *Vibrio alginolyticus* , employ specialised swimming strategies, propelled by a single polar flagellum; they alternate between rapid runs and sharp 180° reversals or 90° ‘flicks’ (Son et al. 2016), conferring advantages in nutrient foraging within oligotrophic seawater. Despite their high motility, marine bacteria adopt slower swimming speeds when tracking small phytoplankton, which prolongs the sensory integration time and improves search efficiency (Foffi et al. 2024).

**FIGURE 3:**
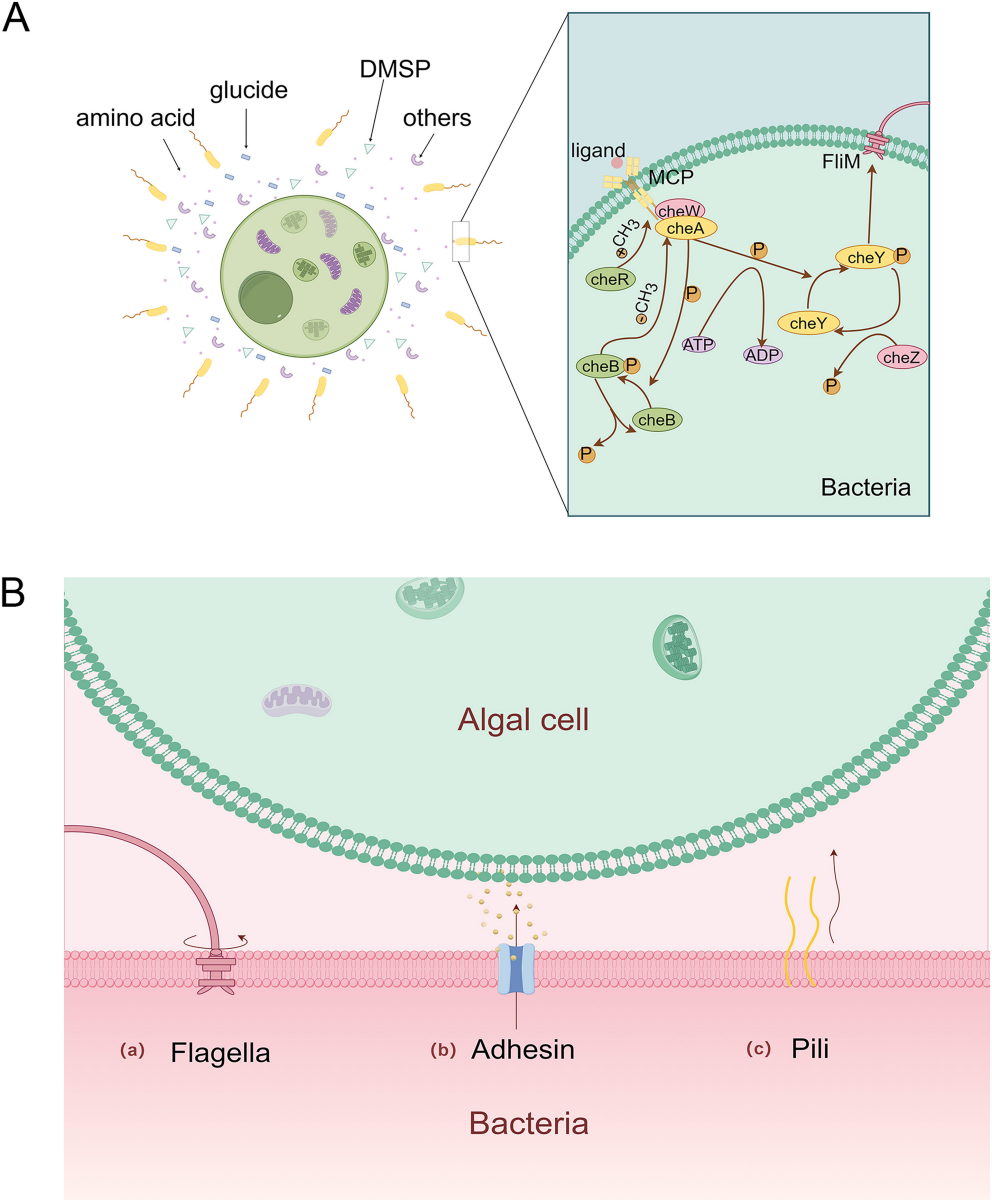
(A) Algicidal bacteria migrate towards algal cells via the canonical chemotaxis pathway. (B) Bacterial attachment to algal cells is mediated through flagella, adhesins, and pili.

In the classical *E. coli* chemotaxis paradigm, extracellular ligands are detected by MCPs (Parkinson 2015), which activate the CheA/CheY two‐component system to modulate flagellar rotation and thus swimming direction. In nutrient‐poor marine environments, bacteria have evolved an expanded repertoire of MCPs, enhancing their adaptive capacity (Lacal et al. 2010). Notably, the genomes of algicidal *Roseobacter* clade (MRC) bacteria typically encode c‐di‐GMP signalling systems, with over half of MRC genomes containing genes for motility, chemotaxis and diverse chemoreceptors, likely facilitating algal localization and stable interspecies interactions (Dang and Lovell 2016) (Figure 3A).

Chemotaxis is also implicated in the initial stages of bacterial surface colonisation. For instance, deletion of the CheA/CheB two‐component system in *Marinobacter adhaerens* HP15 significantly reduced its attachment to diatoms (Sonnenschein et al. 2012). Similarly, non‐motile or morphologically altered mutants of *Silicibacter* sp. TM1040 exhibited impaired attachment to dinoflagellates (Miller and Belas 2006). Once chemotactic bacteria enter the phycosphere and attach to phytoplankton cells (Mayali et al. 2011) or extracellular matrices, they can maintain proximity to their targets and maximise algicidal activity (Seymour et al. 2009, 2008).

### 4.2 Bacterial Attachment to Algal Cells and the Formation of Bacterial–Algal Biofilm Consortia

Attachment represents a critical step in the direct algicidal activity of bacteria, as it prolongs the period of interaction between bacterial and algal cells. Live‐cell imaging combined with scanning and transmission electron microscopy has directly visualised the attachment of algicidal bacterium GD3 and *V. coralliilyticus* to the cell membrane of *K. mikimotoi* cells (Shi et al. 2023; Yu et al. 2024). Depending on their lifestyle, bacteria can be classified as particle‐attached or free‐living (Shi et al. 2023), and field surveys have shown that the abundance of particle‐attached bacteria with algicidal activity in diatom blooms is approximately fivefold higher than that of free‐living counterparts (Park et al. 2010). In co‐culture experiments, the number of attached bacteria per diatom cell is strongly and positively correlated with the rate of algal lysis (Cai et al. 2023). Furthermore, the addition of organic amendments that enhance bacterial adhesion significantly increases algal lysis rates. Collectively, these observations underscore the pivotal role of bacterial adhesion in mediating algicidal effects. The algicidal bacteria, through their adhesion to algal cells, not only prolong the duration of their interaction (Slightom and Buchan 2009), but may also serve as a prerequisite for the algicidal effect. In some cases, adhesion is not merely advantageous but may be essential. For instance, *Alteromonas* sp. L15 requires adhesion to the diatom surface to sense algal surface‐derived chemical cues that activate extracellular proteases capable of degrading frustulin, thereby enabling algal cell lysis (Cai et al. 2023).

The molecular mechanisms underlying bacterial adhesion to algal cells remain incompletely understood, but both flagella and pili appear to be important mediators. In the case of the chitinase‐producing strain LY03 interacting directly with the diatom *T. pseudonana* , bacteria were observed to attach to algal surfaces via flagella before secreting chitinases to degrade the algal cell wall, ultimately leading to cell rupture (Li, Lei, et al. 2016). This process may be regulated by a two‐component system, as *Pseudoalteromonas piscicida* has been shown to modulate chitinase gene expression via the CdsS/CdsR pathway following surface colonisation (Miyamoto et al. 2007). Metagenomic surveys of bacteria during bloom events dominated by diatoms have identified tad gene clusters associated with tight adhesion, suggesting that these clusters encode pili enabling firm attachment to diatoms (Isaac et al. 2021). Beyond adhesion, pili have also been implicated in virulence regulation, as seen in *Pseudomonas aeruginosa* (Persat et al. 2015). Some members of the phylum *Bacteroidota*, which lack flagella, may attach to algal surfaces through stochastic encounters and subsequently move across the cell surface via gliding motility (Buchan et al. 2014). Although the precise mechanisms of gliding remain elusive, type IV pili, extracellular polysaccharides (EPS) and adhesins are likely involved (McBride 2001). Genomic analyses of the gliding bacterium *Flavobacterium* have identified adhesins with conserved peptide motifs, cadherins and bundled filament proteins, all of which may contribute to adhesion (Woyke et al. 2009) (Figure 3B).

Typically, particle‐associated bacteria produce extracellular polymeric substances (EPS), adhesins and other matrix components upon attachment to organic matter, linking individual cells into structured microbial aggregates known as biofilms (O'Toole et al. 2000). These biofilms possess interconnected channels that facilitate the exchange of nutrients, water and gases, thereby optimising resource use and conferring resistance to shear forces in aquatic environments (Bartual et al. 2017) (Steele et al. 2014). In bacterial–algal interactions, initial bacterial attachment to algal surfaces can be reinforced through the reciprocal stimulation of extracellular product secretion, resulting in bacterial–algal consortia with enhanced adhesion and algicidal efficiency. It has been demonstrated in the model system involving the marine coccolithophore *E. huxleyi* and the algicidal bacterium *Phaeobacter inhibens* (Lipsman et al. 2024) that succinate, secreted by algal cells, may act as a chemical signal that promotes bacterial secretion of EPS. This signalling enhances bacterial adhesion and facilitates EPS release, which in turn stimulates the production of transparent exopolymer particles (TEP) by the algal cells. The formation of TEP contributes to the establishment of an extracellular matrix (ECM), thereby promoting the formation of a distinct algal‐bacterial consortium. In such consortia, bacteria are better equipped to exert their algicidal effects. Moreover, other studies suggest that IAA secreted by *Vibrio* species (Gutierrez et al. 2009) may play a role in stimulating the mucus exudation of marine algae and in the formation of Vibrio‐algal consortia (Mazur and Homme 1993). Once attached to algal cell surfaces, bacteria can enrich IAA concentrations, and at high levels, IAA exhibits potent algicidal activity (Segev et al. 2016).

### 4.3 Role of QS in Algicidal Processes

QS is a specialised intra‐ and interspecific communication mechanism among bacteria, enabling population density‐dependent perception, signal transduction and adaptive responses. Canonical QS pathways are characterised by the production, release and detection of low‐molecular‐weight signalling molecules, collectively termed autoinducers, which, upon reaching threshold concentrations indicative of sufficient population size, trigger coordinated behaviours (Dang and Lovell 2016).

**FIGURE 4:**
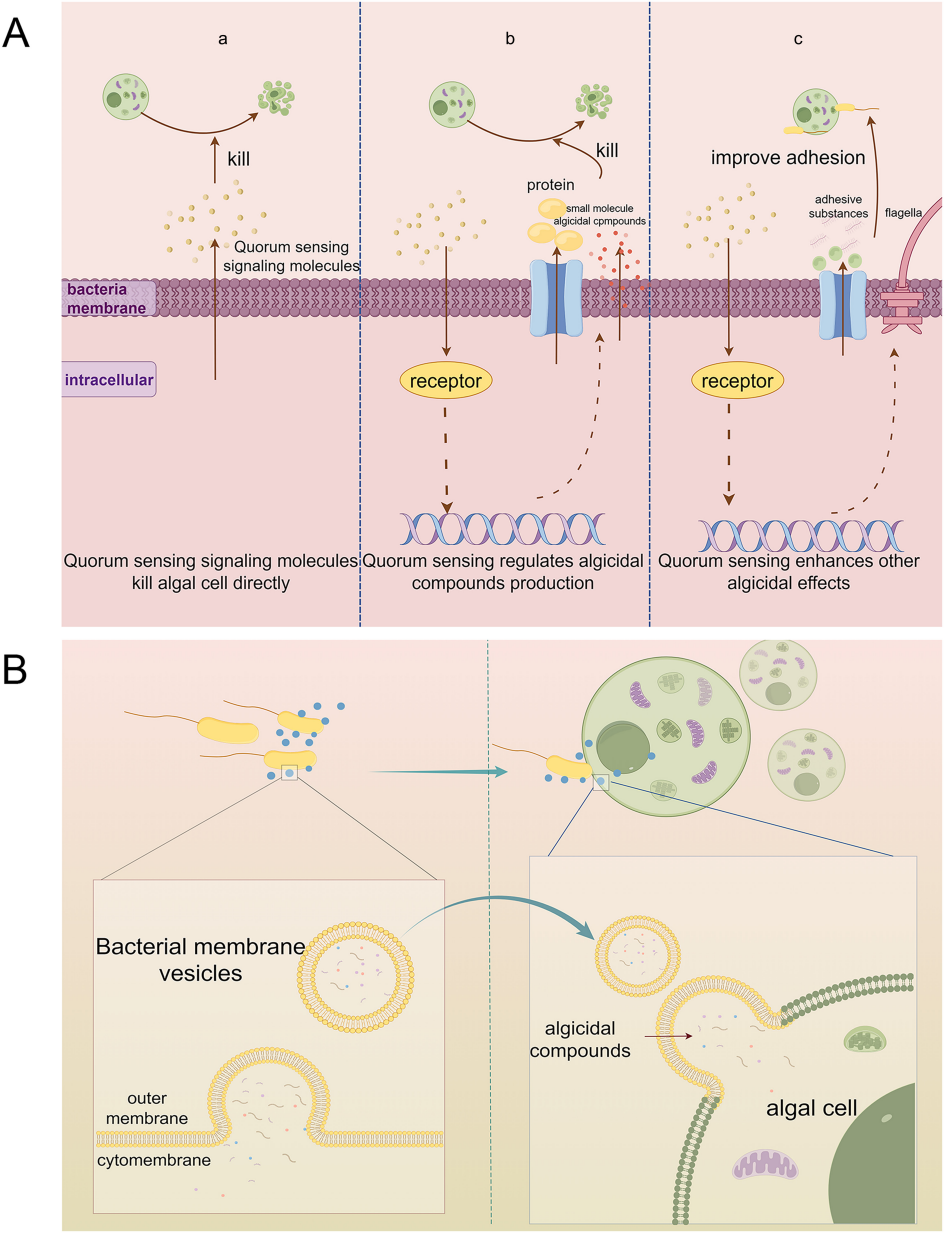
(A) The role of bacterial quorum sensing in algal killing (a). Quorum‐sensing signals in bacteria directly induce cytotoxicity in algal cells. (b) These signals orchestrate the biosynthesis of algicidal metabolites. (c) Quorum‐sensing pathways modulate extracellular polysaccharide secretion and flagellar function, strengthening bacterial adhesion and promoting algal cell demise. (B) Bacterial delivery of algicidal substances via vesicle‐mediated transport.

In recent years, the role of QS in bacterial algicidal activity has attracted increasing attention. Certain marine bacteria produce small‐molecule compounds that act as QS signals. Within the phylum *Proteobacteria*, cell–cell communication is commonly mediated by AHLs, encompassing both short‐chain and long‐chain variants. Short‐chain AHLs include *N*‐hexanoyl‐l‐homoserine lactone (C6‐HSL), *N*‐3‐hydroxyhexanoyl‐l‐homoserine lactone (OH‐C6‐HSL) and *N*‐3‐oxohexanoyl‐L‐homoserine lactone (oxo‐C6‐HSL), while long‐chain AHLs include *N*‐tetradecanoyl‐L‐homoserine lactone (C14‐HSL) and *N*‐3‐hydroxytetradecanoyl‐l‐homoserine lactone (Stock et al. 2020). These QS molecules contribute to algicidal activity through three primary mechanisms. First, some act directly as algicidal agents. For example, *Pseudomonas piscicida* releases 2‐heptyl‐4‐quinolone (HHQ), which induces mortality in *E. huxleyi* (Harvey and Whalen 2016), potentially by disrupting electron transport in the photosynthetic system (Dow et al. 2020). In diatom‐focused studies, short‐chain AHLs generally did not affect growth, whereas certain long‐chain AHLs exhibited inhibitory or lethal effects. Transcriptomic analyses suggest that these long‐chain AHLs may impede growth by modulating lipid metabolism and cell cycle progression (Stock et al. 2020). Evidence exists that bacterial quorum‐sensing molecules alter the composition and abundance of lipids in algal cells (Parveen and Patidar 2023). Second, QS enables coordinated regulation of diverse algicidal compounds (Dow 2021). In *Aeromonas* sp. GLY‐2107, the production of two algicidal metabolites is regulated by short‐chain AHLs, with C4‐HSL serving as a key signal. Disruption of the *agyI* AHL synthase gene abolished production of these compounds (Guo et al. 2016). Similarly, the AI‐2 signal of *Deinococcus* sp. Y35 markedly enhances the synthesis of algicidal pigments, increasing its algicidal potency (Han et al. 2023). Third, QS molecules can enhance algicidal efficiency by modulating bacterial motility and adhesion, thereby improving colonisation of the phycosphere. QS signalling has been shown to suppress motility while promoting adhesion via flagellar regulation (Fei et al. 2020). During their investigation of the phycosphere microecosystem associated with the diatom *Asterionellopsis glacialis* , Fei et al. (2020) demonstrated that bacterial attachment to algal cells is restricted to taxa equipped with a fully functional quorum‐sensing system. The QS molecule 3‐oxo‐C16:1‐HSL strongly inhibited bacterial motility and promoted attachment, and metagenomic analyses have detected increased AHL concentrations during bacterial colonisation of algal cells (Figure 4A) (Isaac et al. 2021).

The genetic basis of QS‐mediated algicidal activity varies across taxa. In *Bacillus*, the NprR/NprX system mediates QS‐dependent regulation of high‐molecular‐weight algicidal compounds against *M. aeruginosa* , with gene disruption leading to reduced activity (Wu et al. 2017). In Gram‐negative bacteria, loss of *luxS* results in decreased algicidal compound production and diminished activity (Liu et al. 2022).

Numerous studies have reported a positive correlation between algicidal efficacy and bacterial density (F. Liu 2023; Jia, Cheng, et al. 2023; Shi et al. 2013). Among the most extensively investigated algicidal bacteria are genera such as *Pseudomonas* sp., *Pseudoalteromonas* sp., *Vibrio* sp. and *Alteromonas* sp., whose algicidal activity is often regulated by QS systems (Kahla et al. 2021). The activation of QS is closely linked to bacterial density: as cell density increases, the concentration of signalling molecules rises until a threshold is reached, triggering receptor activation and downstream gene expression (Williams et al. 2007). Therefore, in studies of QS‐mediated algicidal bacteria, bacterial concentration is likely a key factor influencing their algicidal capacity (Shi et al. 2013). However, it remains to be conclusively demonstrated whether QS‐dependent algicidal activity is strictly density‐dependent. Thus, further research to elucidate the interplay among bacterial density, QS activation and algicidal function will be crucial for advancing our mechanistic understanding and practical application of bacterial agents in algal bloom control.

### 4.4 Bacterial Delivery of Algicidal Compounds via Extracellular Vesicles

Extracellular vesicles (EVs) are membrane‐bound structures secreted into the extracellular milieu, enriched with proteins, nucleic acids, signalling molecules and toxins. They have been identified in bacteria, archaea and eukaryotes (Di Naro et al. 2025), functioning as mediators of intercellular communication. In recent years, EVs have attracted considerable attention, with most studies focusing on mammalian cells and model bacterial species; however, their ecological roles in aquatic systems are increasingly recognised (Schatz 2018). Algal cells can secrete vesicles as an early response to viral infection. Upon infection, algae release large quantities of vesicles that encapsulate viral particles, thereby prolonging viral half‐life in the environment and increasing the risk of infection. When these infection‐derived vesicles are internalised by healthy algal cells, they enhance the cells' susceptibility to subsequent viral attack, resulting in accelerated lysis and higher viral yields. This process sustains effective viral infection and transmission within the population and can contribute to the termination of algal blooms (Schatz et al. 2017).

Bacterially derived EVs also play a pivotal role in algicidal interactions. For instance, outer membrane vesicles of the coral pathogen *Vibrio shilonii* have been found to contain quorum‐sensing signal molecules such as AHL (Li, Azam, et al. 2016), Given the close association between coral disease and its symbiotic dinoflagellates (*Symbiodinium*) (Banin et al. 2001), it is plausible that bacteria may influence coral health by delivering vesicle‐contained factors that adversely affect these symbionts. Moreover, bacterial EVs can directly transport algicidal compounds. Recent work on *Chitinimonas* sp. LY03 revealed that its membrane vesicles exhibit algicidal activity comparable to that of the producing bacterial strain. Purification and structural elucidation via nuclear magnetic resonance spectroscopy and high‐resolution mass spectrometry identified the active agent as a pyrrole‐core macrolactam belonging to the tambjamine antibiotic family, designated Tambjamine LY2. Microscopic imaging confirmed that LY2 enters *Heterosigma akashiwo* and *T. pseudonana* cells via membrane fusion. These findings demonstrate that bacterial vesicles can encapsulate and efficiently deliver hydrophobic algicidal metabolites to algal targets, opening a new chapter in our understanding of bacteria–algae interactions (Figure 4B) (Li, Wang, et al. 2024).

## 5 Mechanisms of Algal Cell Death

Algal cells collapse and lyse under the stress imposed by algicidal substances secreted by bacteria, releasing their intracellular contents and thereby supplying nutrients to the bacteria. The dominant mechanisms by which different algicidal compounds induce algal cell death may differ (Yang et al. 2025). Current evidence indicates that bacterial induction of algal cell death predominantly occurs through disruption of cell membrane integrity, impairment of photosynthesis, induction of oxidative stress and perturbation of calcium homeostasis.

### 5.1 Loss of Cell Integrity

Algicidal compounds produced by bacteria can act on the constituents of algal cell walls and plasma membranes, leading to cell lysis. For example, chitinase secreted by *Chitinophaga* sp. can degrade chitin, which is a major constituent of diatom cell walls (Li, Lei, et al. 2016). In addition, some bacteria secrete beta‐glucosidase and other glycosidases as algicidal agents. Their targets may lie in the cell wall. It has been reported that, in coculture with *Prorocentrum micans* , *Alteromonas* sp. exhibit increased beta‐glucosidase gene expression, while the alga's cell‐wall polysaccharide content declines, providing a potential piece of evidence for this mechanism (Shi et al. 2018). Marine bacterium *Flammeovirga yaeyamensis* shows activities of amylase, cellulase and xylanase, which are associated with the degradation of algal cell walls (Chen et al. 2013). Some small‐molecule algicidal compounds can cross the cell membrane into the cytoplasm, causing cytosolic acidification over time (Lu et al. 2016), potentially triggering ROS production and lipid peroxidation, ultimately leading to intracellular dissolution of algal cells. Beyond targeting cell‐wall and membrane components, certain algicidal substances can alter ion permeability, resulting in cell rupture (Jeong and Son 2021). For instance, violacein produced by *Chromobacterium violaceum* can rapidly disrupt algal cell permeability, provoking prolonged oxidative stress and causing algal cell rupture and death (Cai et al. 2024).

### 5.2 Photosynthetic Uncoupling and Inhibition of Photosystem Activity

Photosynthetically fixed solar energy is the primary energy source for unicellular phytoplankton, and damage to the photosynthetic apparatus can directly lead to cell death (Li, Chen, et al. 2024). Chlorophyll A, a key pigment of the photosynthetic system, not only reflects the photosynthetic state of algal cells but is also frequently used as an indicator of algal mortality in many bacterium‐derived algicidal studies (Zhang et al. 2024; Li, Chen, et al. 2024; Liu et al. 2024). When the photosynthetic system is inhibited or disrupted, chlorophyll A content, photosynthetic efficiency and electron transport rate all decline markedly (Jia, Lu, et al. 2023), indicating that the cells are near collapse.

Certain algicidal compounds act by perturbing the electron transport chain within the photosynthetic apparatus to suppress photosynthetic activity. *Sphingomonas* sp. M‐17 produces argimycin A, which can selectively inhibit cyanobacterial growth; studies suggest this may disrupt the function of the phycobilisome within PSII, preventing energy transfer from the phycobilisome to chlorophyll A and thereby affecting light‐energy conversion (Hibayashi and Imamura 2003). In addition, algicidal substances secreted by *Bacillus subtilis* , such as 4‐acetamido‐butanoic acid (4‐ABC) and 8‐hydroxyquinoline (8‐HQL), have been shown, via measurements of chlorophyll fluorescence, to suppress photosystem activity in *M. aeruginosa* . They reduce photosynthetic parameters Fv/Fm, ѱ_0_ and φ_E0_ (the PSII photochemical efficiency and the electron transport efficiency beyond QA), while increasing the energy‐dissipation quantum yield φD0, indicating these compounds hinder PSII electron transfer and compromise photosynthetic performance (Liu et al. 2024). The 2‐MECHD compound produced by *Pseudomonas fragi* YB2, which resembles an acetylacetonate structure capable of chelating metals, suggests that its target within the photosynthetic electron transport chain may be non‐heme iron in PSII, plastoquinone (PQ) and the iron–sulphur cluster in ferredoxin (Fd) (Zhang et al. 2024). Further, the cytochrome b6f complex has been identified as a major binding site for 2‐alkyl‐4‐quinolones (HHQ) isolated from algicidal marine bacteria (Dow et al. 2020).

Beyond action on the electron transport chain, some algicidal compounds can uncouple the algal photosynthetic system, altering the outcome of photosynthesis. For example, a marine *Vibrio* sp. (*Vibrio shilo*i) (Banin et al. 2001) produces a linear proline‐rich 12‐mer peptide toxin P that, in the presence of ammonium ions, inhibits photosynthesis in a dinoflagellate, likely by promoting ammonium uptake and perturbing the cellular proton gradient, thereby causing photosynthetic uncoupling. The photosynthetic system of algal cells operates as a tightly integrated, sensitive machine; disruption of any link can derail energy conversion, and given its central role in algal survival, the photosynthetic apparatus represents a favourable target for algicidal strategies.

### 5.3 Oxidative Stress and ROS Generation

ROS refer to hydrogen peroxide, superoxide anion, singlet oxygen, hydroxyl radicals and other species generated during electron transfer in photosynthesis and respiration as well as in other enzymatic reactions. These molecules are highly oxidising, and under normal conditions cellular antioxidant enzymes and antioxidants promptly detoxify them to maintain redox balance. When cells face environmental stress, ROS production increases. If not promptly scavenged, accumulated ROS can attack cellular structures, including DNA and proteins, promote lipid peroxidation and compromise membrane integrity, ultimately leading to cell death (Fulda et al. 2010; Mallick and Mohn 2000).

ROS generation is a common phenomenon when algal cells are subjected to algicidal bacteria or algicidal substances (Li et al. 2015; Lin et al. 2023; Zhang et al. 2014). This may reflect multi‐factorial causes: ROS production can arise as part of algal defence responses (Low and Merida 1996), or result from damage to cellular structures caused by algicidal compounds, such as impairment of photosynthetic systems that elevates ROS (Zhang et al. 2014). The algal antioxidant system comprises enzymatic antioxidants (SOD, CAT, APX and POD) and non‐enzymatic antioxidants (ascorbate, glutathione and carotenoids), which are commonly used to assess the oxidative stress status of algal cells (Mallick and Mohn 2000). Malondialdehyde (MDA) content is another important parameter for judging oxidative stress, as MDA is one of the terminal products of lipid peroxidation and reflects the extent of membrane lipid oxidative damage (Tsikas 2017). For example, in the indirect algicidal action of *Bacillus subtilis* on *Spirogyra gracilis*, the contents of MDA, POD, CAT and SOD increase, indicating that the algicidal compounds induce oxidative damage in the algal cells (Gu et al. 2024). Prolonged exposure to stress can lead to algal apoptosis. In diatom cells subjected to stress from the algicidal bacterium *Bacillus mycoides* , cysteine proteases involved in programmed cell death were activated, which can scavenge ROS and attenuate the ROS‐induced upregulation of glutathione synthetase expression; concurrently, the death‐specific protein (DSP), implicated in transcriptional regulation of photosynthesis, also increased. These findings suggest that diatoms employ mechanisms to neutralise excess ROS under oxidative stress while concurrently initiating programmed cell death (Bayramova et al. 2024). Other work suggests that excessive ROS produced by *K. mikimotoi* under stress can promote apoptosis by stimulating caspase‐3 (Han et al. 2018).

### 5.4 Disruption of Calcium Homeostasis

Calcium ions (Ca^2+^) act as pivotal signalling molecules in a wide range of cellular processes, including maintaining the stability of the cell wall and plasma membrane, regulating cell proliferation and modulating metabolic activity (Bagur and Hajnóczky 2017). In studies on algicidal bacteria targeting *Chlamydomonas reinhardi*, it was found that both pyrrolnitrin and pyoluteorin secreted by *Pseudomonas protegens* perturb intracellular Ca^2+^ dynamics. Exposure of algal cells to 20 μM pyrrolnitrin induced a rapid and pronounced intracellular Ca^2+^ surge, while treatment with 100 μM pyoluteorin similarly elevated Ca^2+^ levels, triggering explosive cell death. Permeability assays suggested that this interference with algal Ca^2+^ homeostasis may occur via a mechanism independent of membrane rupture (Rose et al. 2021). Further evidence was obtained in the interaction between *P. fragi* YB2 and *Chlorella*, where non‐invasive micro‐test technology (NMT) was used to directly quantify cytosolic Ca^2+^ fluxes without damaging algal cells. Application of YB2 supernatant significantly increased Ca^2+^ influx in *Chlorella*, disrupting intracellular calcium homeostasis (Zhang et al. 2024).

### 5.5 Other Mechanisms of Algal Cell Death

Beyond the commonly described algal cell death mechanisms, other death pathways have been explored. For example, algicidal substances can affect the cell cycle to induce programmed cell death in algae (Wang and Coyne 2023), and death can also result from inhibition of algal cell division (Li, Lin, et al. 2014; Van Tol et al. 2017). In the transcriptome of *H. akashiwo* exposed to *Pseudalteromonas* sp. LD‐B1, upregulation of autophagy‐related genes such as the ubiquitin‐like modifier‐activating enzyme (ATG7), Beclin (BECN) and the 5′‐AMP‐activated protein kinase (PRKAA) was observed, indicating an autophagic response in the algal cells (Xu, Chen, et al. 2024). Additionally, during *Microbulbifer* sp.‐mediated killing of *Phaeocystis globosa* , the algal cells may undergo autophagic cell death (Zhu et al. 2022).

**FIGURE 5:**
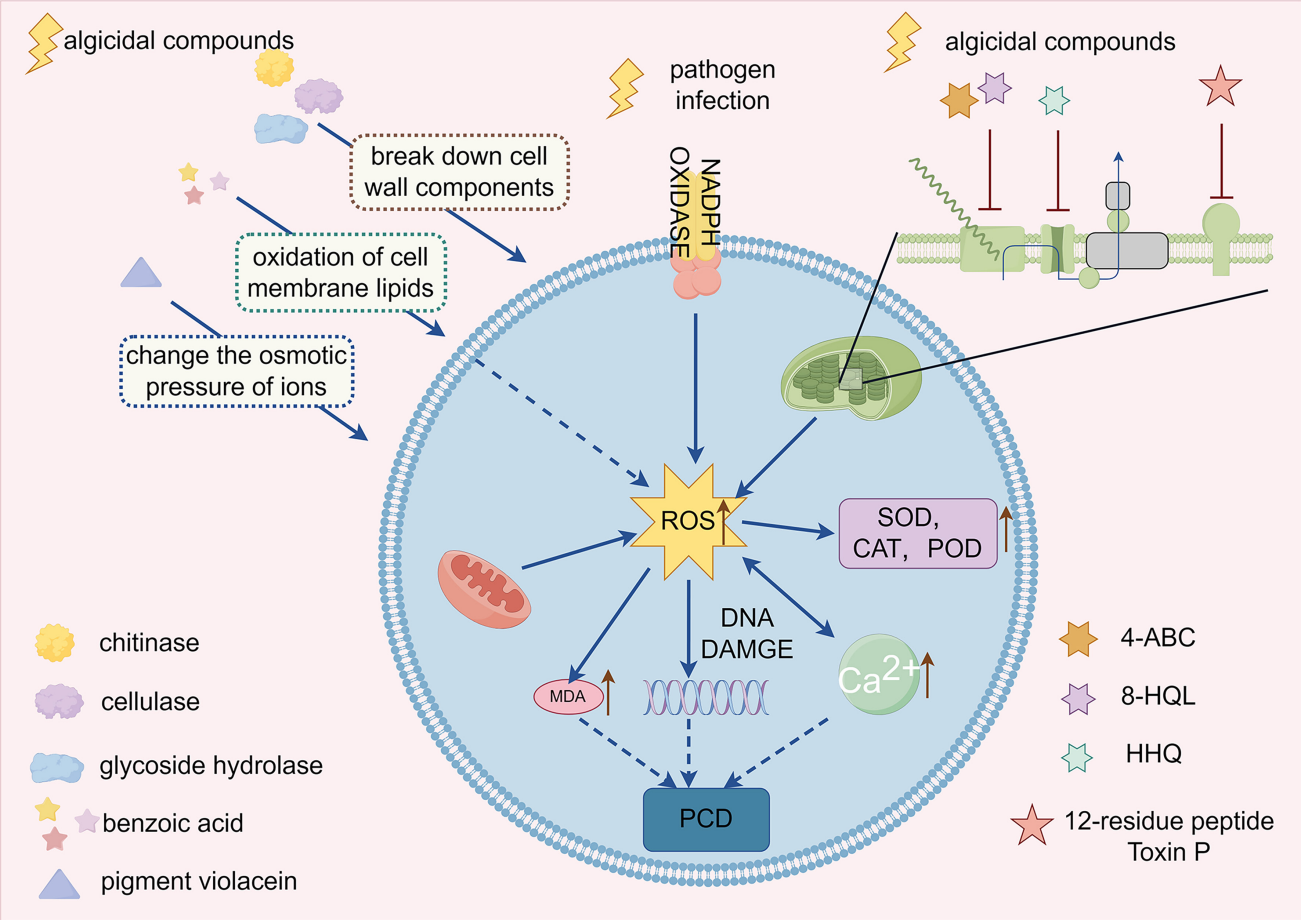
Mechanisms by which bacteria and their algicidal compounds induce algal cell death: Bacteria can disrupt the integrity of extracellular structures and impair algal photosynthesis. Algal defence responses trigger a substantial increase in reactive oxygen species (ROS), which elevate intracellular Ca^2+^ concentrations and activate programmed cell death in the algal cells.

Overall, algal cell death is a complex and multifaceted process. It can arise not only from direct lysis inflicted by algicidal substances but also from disruption of normal cellular physiology that triggers programmed cell death. ROS play a central role as key signalling mediators in death regulation. Mechanical damage, impairment of photosynthesis (Pospíšil 2012), and upregulation of intracellular Ca^2+^ caused by algicidal bacteria can all provoke ROS production; likewise, under stress, excessive ROS accumulation damages organelles and biomolecules, forcing the cell to choose between survival and death (Zhang et al. 2020). ROS can regulate downstream responses via eight distinct pathways to either rescue the cell or induce death (Tang et al. 2025). ROS not only act as signals of programmed cell death alongside elevated Ca^2+^, but ROS‐induced lipid peroxidation products also participate in death induction (Aguilera et al. 2022), and ROS is linked to autophagic death as well (Pérez‐Pérez et al. 2012). The modes of death are intricately interconnected; cumulative interactions lead to cellular collapse, and the dominant mode may be determined by the specific algicidal substance and the algal species involved. Therefore, when investigating algal death mechanisms, identifying the principal sites of action may be the most critical aspect to emphasise (Figure 5).

## 6 Discussion

As researchers screen for bacteria with high algicidal activity from the phycosphere, notable progress has been achieved using algicidal bacteria to mitigate blooms. *Bacillus subtilis* S4, for example, can lyse *M. aeruginosa* with an algicidal kill rate of up to 98%, and about 89% of the released microcystins are degraded by the bacteria, suggesting potential for remediation of nutrient‐rich waters experiencing blooms (Chen et al. 2024). Similarly, *Paenibacillus* sp. can reduce toxin concentrations by approximately 90% when targeting *M. aeruginosa* (Jia, Cheng, et al. 2023). In addition, *Pseudomonas* sp. Ps3 demonstrates substantial bloom‐dissolving activity against the dinoflagellate *Gymnodinium catenatum* and *K. mikimotoi*, achieving algicidal rates of 83.0% and 78.3%, respectively (Zheng et al. 2023). Nevertheless, practical deployment of algicidal bacteria faces several challenges. In laboratory co‐culture systems, confined conditions allow bacteria to exert strong algicidal effects, but environmental variability in natural settings may compromise efficacy. *Pseudomonas fluorescens* SK09 is the most frequently studied algicidal bacterium in both laboratory studies and field surveys and can effectively control harmful blooms, and it exhibits robust, species‐specific algicidal activity against *Stephanodiscus hantzschii* in vitro (Jung et al. 2010). However, field trials show a significant reduction in algicidal activity (Noh et al. 2017). Moreover, the life state of algal cells can differ under varying growth conditions; for instance, *M. aeruginosa* tends to occur as colonies in natural waters but as single cells under laboratory culture, a difference driven by the production of extracellular polymers by associated bacteria that promotes aggregation in situ (Wang et al. 2016). Beyond inconsistent efficacy, safety and contamination concerns must also be addressed: do algicidal preparations affect non‐target organisms, and do the bacterial cultures leave residual nutrients or other contaminants (F. Liu 2023)? Additionally, can the toxins released from lysed cells be reliably removed or purified in practice? These considerations underscore the need for careful ecological risk assessment and the development of field‐relevant strategies before any widespread application.

As these challenges drive deeper investigation, we recognise that bacteria–algae interactions in natural settings encompass three main relationships: symbiosis, competition and antagonism. These interactions are largely governed by nutrient acquisition and may shift with environmental conditions. Algicidal bacteria can suppress algal cells through direct contact or via secreted active compounds. Direct‐contact killing may require bacterial appendages, such as flagella or pili, to deliver attack or may be triggered only when algal surface–released cues induce the production of active algicidal agents. Direct contact also shortens the effective distance and elevates local algicidal concentrations, thereby enhancing efficacy. Indirect algicidal action relies on diffusible algicidal substances; constitutive compounds are produced irrespective of algal exudates, whereas inducible compounds are generated only after sensing algal‐derived signals. In aquatic environments, bacteria detect algal attractants through MCPs to modulate flagellar activity and execute chemotaxis into the phycosphere, a key prerequisite for in situ algicidal activity. Adhesion following chemotaxis represents further engagement with the algal cell and is necessary for some direct‐contact algicidal strains; bacterial flagella, pili and secreted adhesins likely facilitate this adhesion, potentially under quorum‐sensing control. Moreover, quorum‐sensing signals themselves can act as algicidal molecules or serve as switches for algicidal gene expression. Emerging work indicates that bacterial vesicles can function as cargo vectors for algicidal substances, broadening the modes of bacterial algicidal action and revealing important facets of alga–bacteria communication. Under exposure to algicidal bacteria or substances, algal cell walls or membranes may be degraded, leading to cell lysis; damage to the photosynthetic apparatus triggers oxidative stress, and accumulated ROS oxidise membrane lipids and DNA, causing cellular injury. Calcium ions and ROS often act synergistically as signals that coordinate the death process.

Despite presenting a refined description of algicidal bacteria, this field lacks the depth typical of mechanistic studies in bacteria–host interactions elsewhere, highlighting the necessity for rigorous, high‐quality investigations to uncover the underlying mechanisms. In recent years, the maturation and cost reduction of omics technologies have led to an increasing number of studies employing multi‐omics approaches to investigate the molecular mechanisms of bacteria‐algae interactions, a development that could significantly aid future research. For instance, in the interaction between *Brevibacillus laterosporus* and *M. aeruginosa* , bacterial transcriptome sequencing following co‐culture indicated that bacteria may degrade amino acids and fatty acids as sources of ATP and energy, while membrane transport proteins secrete hydrolases and proteases into the external environment to disrupt algal cells, leading to cell death and achieving algicidal effects (Zhang et al. 2022). Jiaying Yu et al. through transcriptomic analysis of bacterial‐algal interactions not only explored the algicidal mechanisms of the direct killing bacterium *V. coralliirubri* against *K. mikimotoi* but also provided clues regarding the underlying mechanisms of algal cell death (Yu et al. 2024). Similarly, Lee et al. (2024) utilised tandem mass spectrometry‐based proteomics to investigate the interaction between the algicidal bacterium *Maribacter dokdonesis* P4 and the red tide algae *K. mikimotoi*. They found that bacterial stress led to the downregulation of key mitochondrial and chloroplast proteins in the algae, which inactivated their functions, ultimately resulting in cellular oxidative stress and cell death. Taken together, the rapid development of multi‐omics approaches provides a powerful toolkit for dissecting the molecular mechanisms of bacterium–algae interactions. However, omics‐derived insights must be validated by targeted molecular biology experiments, and current limitations in algal genome annotation, particularly the relatively low annotation rates among dinoflagellates (Yang et al. 2010; Lin 2024), remain a key bottleneck. Looking ahead, improvements in genome annotation methods, the development of more precise omics analyses, and rigorous cross‐disciplinary validation will help to delineate the mechanisms of bacterium–algae interactions more comprehensively and provide a more robust theoretical framework and technical support for the biocontrol of red tides.

## Author Contributions

**Jiaxin Wang:** conceptualization, writing – original draft. **Binfu Xu:** writing – review and editing. **Lixing Huang:** conceptualization, funding acquisition, writing – review and editing.

## Funding

This work was supported by the Natural Science Foundation of Fujian Province (No. 2022J02044).

## Conflicts of Interest

The authors declare no conflicts of interest.

## Data Availability

Data sharing not applicable to this article as no datasets were generated or analysed during the current study.

## References

[emi470305-bib-0001] Aguilera, A. , A. Distéfano , C. Jauzein , et al. 2022. “Do Photosynthetic Cells Communicate With Each Other During Cell Death? From Cyanobacteria to Vascular Plants.” Journal of Experimental Botany 73: 7219–7242. 36179088 10.1093/jxb/erac363

[emi470305-bib-0002] Amin, S. A. , D. H. Green , and M. C. Hart . 2009. “Photolysis of Iron–Siderophore Chelates Promotes Bacterial–Algal Mutualism.” Proceedings of the National Academy of Sciences 106: 17071–17076. 10.1073/pnas.0905512106. PMC2761308 19805106

[emi470305-bib-0003] Amin, S. A. , L. R. Hmelo , H. M. Van Tol , et al. 2015. “Interaction and Signalling Between a Cosmopolitan Phytoplankton and Associated Bacteria.” Nature 522: 98–101. 26017307 10.1038/nature14488

[emi470305-bib-0004] Anabtawi, H. M. , W. H. Lee , A. Al‐Anazi , M. M. Mohamed , and A. Aly Hassan . 2024. “Advancements in Biological Strategies for Controlling Harmful Algal Blooms (HABs).” Water 16: 224.

[emi470305-bib-0005] Anderson, D. M. , E. Fensin , C. J. Gobler , et al. 2021. “Marine Harmful Algal Blooms (HABs) in the United States: History, Current Status and Future Trends.” Harmful Algae 102: 101975. 33875183 10.1016/j.hal.2021.101975 PMC8058451

[emi470305-bib-0006] Aoki, K. , H. Kuroda , T. Setou , et al. 2019. “Exceptional Red‐Tide of Fish‐Killing Dinoflagellate Karenia Mikimotoi Promoted by Typhoon‐Induced Upwelling.” Estuarine, Coastal and Shelf Science 219: 14–23.

[emi470305-bib-0007] Azam, F. , and F. Malfatti . 2007. “Microbial Structuring of Marine Ecosystems.” Nature Reviews Microbiology 5: 782–791. 17853906 10.1038/nrmicro1747

[emi470305-bib-0008] Bagur, R. , and G. Hajnóczky . 2017. “Intracellular Ca^2+^ Sensing: Its Role in Calcium Homeostasis and Signaling.” Molecular Cell 66: 780–788. 28622523 10.1016/j.molcel.2017.05.028 PMC5657234

[emi470305-bib-0009] Balaji‐Prasath, B. 2022. “Methods to Control Harmful Algal Blooms: A Review.” Environmental Chemistry Letters 20: 3133–3152. 10.1007/s10311-022-01457-2.

[emi470305-bib-0010] Banin, E. , S. K. Khare , F. Naider , and E. Rosenberg . 2001. “Proline‐Rich Peptide From the Coral Pathogen *Vibrio* *shiloi* That Inhibits Photosynthesis of Zooxanthellae.” Applied and Environmental Microbiology 67: 1536–1541. 11282602 10.1128/AEM.67.4.1536-1541.2001 PMC92766

[emi470305-bib-0011] Barbara, G. M. , and J. G. Mitchell . 2003. “Bacterial Tracking of Motile Algae.” FEMS Microbiology Ecology 44: 79–87. 19719653 10.1111/j.1574-6941.2003.tb01092.x

[emi470305-bib-0012] Bartual, A. , I. Vicente‐Cera , S. Flecha , and L. Prieto . 2017. “Effect of Dissolved Polyunsaturated Aldehydes on the Size Distribution of Transparent Exopolymeric Particles in an Experimental Diatom Bloom.” Marine Biology 164: 120.

[emi470305-bib-0013] Basterretxea, G. , J. S. Font‐Muñoz , and I. Tuval . 2020. “Phytoplankton Orientation in a Turbulent Ocean: A Microscale Perspective.” Frontiers in Marine Science 7: 185.

[emi470305-bib-0014] Bauer, A. , and K. Forchhammer . 2021. “Bacterial Predation on Cyanobacteria.” Microbial Physiology 31: 99–108. 34010833 10.1159/000516427

[emi470305-bib-0015] Bayramova, E. , D. Petrova , A. Marchenkov , et al. 2024. “Differential Expression of Stress Adaptation Genes in a Diatom Ulnaria Acus Under Different Culture Conditions.” International Journal of Molecular Sciences 25: 2314. 38396992 10.3390/ijms25042314 PMC10888605

[emi470305-bib-0016] Bell, W. , and R. Mitchell . 1972. “Chemotactic and Growth Responses of Marine Bacteria to Algal Extracellular Products.” Biological Bulletin 143: 265–277.

[emi470305-bib-0017] Brooks, B. W. , J. M. Lazorchak , M. D. A. Howard , et al. 2016. “Are Harmful Algal Blooms Becoming the Greatest Inland Water Quality Threat to Public Health and Aquatic Ecosystems?” Environmental Toxicology and Chemistry 35: 6–13. 26771345 10.1002/etc.3220

[emi470305-bib-0018] Buchan, A. , G. R. LeCleir , C. A. Gulvik , and J. M. González . 2014. “Master Recyclers: Features and Functions of Bacteria Associated With Phytoplankton Blooms.” Nature Reviews Microbiology 12: 686–698. 25134618 10.1038/nrmicro3326

[emi470305-bib-0019] Cai, G. , X. Yang , X. Yu , W. Zheng , R. Cai , and H. Wang . 2024. “The Novel Application of Violacein Produced by a Marine Duganella Strain as a Promising Agent for Controlling *Heterosigma* *akashiwo* Bloom: Algicidal Mechanism, Fermentation Optimization and Agent Formulation.” Journal of Hazardous Materials 466: 133548. 38262320 10.1016/j.jhazmat.2024.133548

[emi470305-bib-0020] Cai, G. , X. Yu , H. Wang , T. Zheng , and F. Azam . 2023. “Nutrient‐Dependent Interactions Between a Marine Copiotroph *Alteromonas* and a Diatom *Thalassiosira* *pseudonana* .” MBio 14: e00940‐23. 37772817 10.1128/mbio.00940-23 PMC10653928

[emi470305-bib-0021] Cencini, M. , G. Boffetta , M. Borgnino , and F. De Lillo . 2019. “Gyrotactic Phytoplankton in Laminar and Turbulent Flows: A Dynamical Systems Approach.” European Physical Journal E 42: 31. 10.1140/epje/i2019-11792-0 30879226

[emi470305-bib-0022] Chai, W. , Q. Wang , C. Zhao , et al. 2025. “The Effectiveness of Four Algal Technologies in Removing Nutrients and Antibiotics From Biogas Slurry Induced by Different Concentrations of Diethylaminoethyl Hexanoate.” Algal Research 86: 103906.

[emi470305-bib-0023] Chapra, S. C. , B. Boehlert , C. Fant , et al. 2017. “Climate Change Impacts on Harmful Algal Blooms in U.S. Freshwaters: A Screening‐Level Assessment.” Environmental Science & Technology 51: 8933–8943. 28650153 10.1021/acs.est.7b01498

[emi470305-bib-0024] Chen, C.‐Y. , M.‐D. Bai , and J.‐S. Chang . 2013. “Improving Microalgal Oil Collecting Efficiency by Pretreating the Microalgal Cell Wall With Destructive Bacteria.” Biochemical Engineering Journal 81: 170–176.

[emi470305-bib-0025] Chen, Y. , F. Xiong , Y. Zhu , et al. 2024. “A *Bacillus* *subtilis* Strain With Efficient Algaecide of *Microcystis* *aeruginosa* and Degradation of Microcystins.” Frontiers in Microbiology 15: 1430097. 39678917 10.3389/fmicb.2024.1430097 PMC11638172

[emi470305-bib-0026] Clerc, E. E. , J.‐B. Raina , J. M. Keegstra , et al. 2023. “Strong Chemotaxis by Marine Bacteria Towards Polysaccharides Is Enhanced by the Abundant Organosulfur Compound DMSP.” Nature Communications 14: 8080. 10.1038/s41467-023-43143-z PMC10700628 38057294

[emi470305-bib-0027] Coale, T. H. , V. Loconte , K. A. Turk‐Kubo , et al. 2024. “Nitrogen‐Fixing Organelle in a Marine Alga.” Science 384: 217–222. 10.1126/science.adk1075. 38603509

[emi470305-bib-0028] Cole, J. J. 1982. “Interactions Between Bacteria and Algae in Aquatic Ecosystems.” Annual Review of Ecology and Systematics 13: 291–314. 10.1146/annurev.es.13.110182.001451.

[emi470305-bib-0029] Coyne, K. J. , Y. Wang , and G. Johnson . 2022. “Algicidal Bacteria: A Review of Current Knowledge and Applications to Control Harmful Algal Blooms.” Frontiers in Microbiology 13: 871177. 35464927 10.3389/fmicb.2022.871177 PMC9022068

[emi470305-bib-0030] Croft, M. T. , A. D. Lawrence , E. Raux‐Deery , M. J. Warren , and A. G. Smith . 2005. “Algae Acquire Vitamin B_12_ Through a Symbiotic Relationship With Bacteria.” Nature 438: 90–93. 16267554 10.1038/nature04056

[emi470305-bib-0031] Dai, Q. , J. Shan , X. Deng , H. Yang , C. Chen , and Y. Zhao . 2024. “The Characteristics of H6 Against *Microcystis* *aeruginosa* .” Environmental Science and Pollution Research 31: 7702–7711. 38170350 10.1007/s11356-023-31616-z

[emi470305-bib-0032] Dang, H. , and C. R. Lovell . 2016. “Microbial Surface Colonization and Biofilm Development in Marine Environments.” Microbiology and Molecular Biology Reviews 80: 91–138. 10.1128/MMBR.00037-15. 26700108 PMC4711185

[emi470305-bib-0033] Deng, Y. , R. Yu , V. Grabe , et al. 2024. “Bacteria Modulate Microalgal Aging Physiology Through the Induction of Extracellular Vesicle Production to Remove Harmful Metabolites.” Nature Microbiology 9: 2356–2368. 10.1038/s41564-024-01746-2 PMC11371645 39143356

[emi470305-bib-0034] Di Naro, M. , G. Petronio , F. Mukhtar , et al. 2025. “Extracellular Vesicles in Bacteria, Archaea, and Eukaryotes: Mechanisms of Inter‐Kingdom Communication and Clinical Implications.” Microorganisms 13: 636. 40142528 10.3390/microorganisms13030636 PMC11944275

[emi470305-bib-0035] Ding, N. , W. Du , Y. Feng , et al. 2021. “Algicidal Activity of a Novel Indigenous Bacterial Strain of *Paracoccus homiensis* Against the Harmful Algal Bloom Species, *Karenia mikimotoi* .” Archives of Microbiology 203: 4821–4828. 34212209 10.1007/s00203-021-02468-3

[emi470305-bib-0036] Dow, L. 2021. “How Do Quorum‐Sensing Signals Mediate Algae–Bacteria Interactions?” Microorganisms 9: 1391. 34199114 10.3390/microorganisms9071391 PMC8307130

[emi470305-bib-0037] Dow, L. , F. Stock , A. Peltekis , et al. 2020. “The Multifaceted Inhibitory Effects of an Alkylquinolone on the Diatom *Phaeodactylum tricornutum* .” Chembiochem 21: 1206–1216. 31747114 10.1002/cbic.201900612 PMC7217009

[emi470305-bib-0038] Fei, C. , M. A. Ochsenkühn , A. A. Shibl , A. Isaac , C. Wang , and S. A. Amin . 2020. “Quorum Sensing Regulates ‘Swim‐Or‐Stick’ Lifestyle in the Phycosphere.” Environmental Microbiology 22: 4761–4778. 32896070 10.1111/1462-2920.15228 PMC7693213

[emi470305-bib-0039] Feng, L. , Y. Wang , X. Hou , et al. 2024. “Harmful Algal Blooms in Inland Waters.” Nature Reviews Earth & Environment 5: 631–644. 10.1038/s43017-024-00578-2 PMC11849997 39995947

[emi470305-bib-0040] Field, C. B. , M. J. Behrenfeld , J. T. Randerson , and P. Falkowski . 1998. “Primary Production of the Biosphere: Integrating Terrestrial and Oceanic Components.” Science 281: 237–240. 9657713 10.1126/science.281.5374.237

[emi470305-bib-0041] Foffi, R. , D. R. Brumley , F. Peaudecerf , R. Stocker , and J. Słomka . 2024. “Slower Swimming Promotes Chemotactic Encounters Between Bacteria and Small Phytoplankton.” 122: e2411074122. 10.1073/pnas.2411074122. PMC11745318 39792290

[emi470305-bib-0042] Fulda, S. , A. M. Gorman , O. Hori , and A. Samali . 2010. “Cellular Stress Responses: Cell Survival and Cell Death.” International Journal of Cell Biology 2010: 1–23. 10.1155/2010/214074 PMC2825543 20182529

[emi470305-bib-0043] Furusawa, G. , T. Yoshikawa , A. Yasuda , and T. Sakata . 2003. “Algicidal Activity and Gliding Motility of *Saprospira* sp. SS98‐5.” Canadian Journal of Microbiology 49: 92–100. 12718397 10.1139/w03-017

[emi470305-bib-0044] Gallardo‐Rodríguez, J. J. , A. Astuya‐Villalón , A. Llanos‐Rivera , V. Avello‐Fontalba , and V. Ulloa‐Jofré . 2019. “A Critical Review on Control Methods for Harmful Algal Blooms.” Reviews in Aquaculture 11: 661–684.

[emi470305-bib-0045] Garren, M. , K. Son , J.‐B. Raina , et al. 2014. “A Bacterial Pathogen Uses Dimethylsulfoniopropionate as a Cue to Target Heat‐Stressed Corals.” ISME Journal 8: 999–1007. 24335830 10.1038/ismej.2013.210 PMC3996689

[emi470305-bib-0046] Gobler, C. J. 2020. “Climate Change and Harmful Algal Blooms: Insights and Perspective.” Harmful Algae 91: 101731. 32057341 10.1016/j.hal.2019.101731

[emi470305-bib-0047] Gobler, C. J. , O. M. Doherty , T. K. Hattenrath‐Lehmann , A. W. Griffith , Y. Kang , and R. W. Litaker . 2017. “Ocean Warming Since 1982 Has Expanded the Niche of Toxic Algal Blooms in the North Atlantic and North Pacific Oceans.” Proceedings of the National Academy of Sciences of the United States of America 114: 4975–4980. 28439007 10.1073/pnas.1619575114 PMC5441705

[emi470305-bib-0048] Grattan, L. M. , S. Holobaugh , and J. G. Morris . 2016. “Harmful Algal Blooms and Public Health.” Harmful Algae 57: 2–8. 10.1016/j.hal.2016.05.003 PMC5016795 27616971

[emi470305-bib-0049] Griffith, A. W. , O. M. Doherty , and C. J. Gobler . 2019. “Ocean Warming Along Temperate Western Boundaries of the Northern Hemisphere Promotes an Expansion of *Cochlodinium polykrikoides * Blooms.” Proceedings of the Royal Society B: Biological Sciences 286: 20190340. 10.1098/rspb.2019.0340 PMC6571469 31161913

[emi470305-bib-0050] Gu, Y. , H. Wang , H. Cao , et al. 2024. “A Potential Algicidal Bacterium Against Spirogyra Gracilis Blooms: Identification, Algicidal Activity, Algicidal Mode, and Metabolomic Profiling.” Journal of Applied Phycology 36: 3829–3842.

[emi470305-bib-0051] Guo, X. , X. Liu , L. Wu , J. Pan , and H. Yang . 2016. “The Algicidal Activity of *Aeromonas* sp. Strain GLY‐2107 Against Bloom‐Forming *Microcystis aeruginosa* Is Regulated by *N* ‐Acyl Homoserine Lactone‐Mediated Quorum Sensing.” Environmental Microbiology 18: 3867–3883. 27105123 10.1111/1462-2920.13346

[emi470305-bib-0052] Gutierrez, C. K. , G. Y. Matsui , D. E. Lincoln , and C. R. Lovell . 2009. “Production of the Phytohormone Indole‐3‐Acetic Acid by Estuarine Species of the Genus *Vibrio* .” Applied and Environmental Microbiology 75: 2253–2258. 19218411 10.1128/AEM.02072-08 PMC2675210

[emi470305-bib-0053] Han, M. , R. Wang , N. Ding , et al. 2018. “Reactive Oxygen Species‐Mediated Caspase‐3 Pathway Involved in Cell Apoptosis of *Karenia* *mikimotoi* Induced by Linoleic Acid.” Algal Research 36: 48–56.

[emi470305-bib-0054] Han, S. , C. Lu , M. Qin , et al. 2023. “Alleviation Effect of *Deinococcus* sp. Y35 Together With Autoinducer 2 on the Physiological Stress Caused by *Microcystis* *aeruginosa* to Zebrafish Intestines.” Aquaculture 576: 739880.

[emi470305-bib-0055] Han, Y. , N. Jiao , Y. Zhang , et al. 2021. “Opportunistic Bacteria With Reduced Genomes Are Effective Competitors for Organic Nitrogen Compounds in Coastal Dinoflagellate Blooms.” Microbiome 9: 71. 33762013 10.1186/s40168-021-01022-z PMC7992965

[emi470305-bib-0056] Harvey, E. L. , and K. E. Whalen . 2016. “A Bacterial Quorum‐Sensing Precursor Induces Mortality in the Marine Coccolithophore, *Emiliania huxleyi * .” Frontiers in Microbiology 7: 59. 10.3389/fmicb.2016.00059. 26870019 PMC4737879

[emi470305-bib-0057] Hibayashi, R. , and N. Imamura . 2003. “Action Mechanism of a Selective Anti‐Cyanobacterial Compound, Argimicin A.” Journal of Antibiotics 56: 154–159. 12715875 10.7164/antibiotics.56.154

[emi470305-bib-0058] Hu, C. , Z. Shi , T. Hu , Y. Gao , Q. Liu , and C. Wang . 2025. “A Comprehensive Review of Diatom‐Bacterial Interactions Inferred From Bibliometric Analysis.” Reviews in Aquaculture 17: e12974.

[emi470305-bib-0059] Hu, X.‐J. , Y. Xu , H.‐C. Su , et al. 2019. “Algicidal Bacterium CZBC1 Inhibits the Growth of *Oscillatoria* *chlorina* , *Oscillatoria tenuis *, and *Oscillatoria* *planctonica* .” AMB Express 9: 144. 31512077 10.1186/s13568-019-0872-8 PMC6738362

[emi470305-bib-0060] Ianora, A. , M. G. Bentley , G. S. Caldwell , et al. 2011. “The Relevance of Marine Chemical Ecology to Plankton and Ecosystem Function: An Emerging Field.” Marine Drugs 9: 1625–1648. 22131962 10.3390/md9091625 PMC3225939

[emi470305-bib-0061] Isaac, A. , B. Francis , R. I. Amann , and S. A. Amin . 2021. “Tight Adherence (Tad) Pilus Genes Indicate Putative Niche Differentiation in Phytoplankton Bloom Associated Rhodobacterales.” Frontiers in Microbiology 12: 718297. 34447362 10.3389/fmicb.2021.718297 PMC8383342

[emi470305-bib-0062] Jeong, S.‐Y. , and H.‐J. Son . 2021. “Effects of Mycosubtilin Homolog Algicides From a Marine Bacterium, *Bacillus* sp. SY‐1, Against the Harmful Algal Bloom Species *Cochlodinium polykrikoides* .” Journal of Microbiology 59: 389–400. 33779952 10.1007/s12275-021-1086-8

[emi470305-bib-0063] Jia, L. , X. Cheng , L. Fang , and X. Huang . 2023. “Flocculation and Lysis of *Microcystis* *aeruginosa* by Paebubacillus sp. A9 and Inhibition of Microcystin Release.” Environmental Technology & Innovation 31: 103152.

[emi470305-bib-0064] Jia, Y. , J. Lu , M. Wang , et al. 2023. “Algicidal Bacteria in Phycosphere Regulate Free‐Living Symbiodinium Fate via Triggering Oxidative Stress and Photosynthetic System Damage.” Ecotoxicology and Environmental Safety 263: 115369. 37586194 10.1016/j.ecoenv.2023.115369

[emi470305-bib-0065] Jiang, W. , Z. Yu , X. Cao , et al. 2021. “Effects of Soluble Organics on the Settling Rate of Modified Clay and Development of Improved Clay Formulations for Harmful Algal Bloom Control.” Environmental Pollution 289: 117964. 34426199 10.1016/j.envpol.2021.117964

[emi470305-bib-0066] José, M. 2018. “Bacterial Virulence Against an Oceanic Bloom‐Forming Phytoplankter Is Mediated by Algal DMSP.” Science Advances 4: eaau5716. 30397652 10.1126/sciadv.aau5716 PMC6200362

[emi470305-bib-0067] Jung, S. W. , Y.‐H. Kang , T. Katano , et al. 2010. “Testing Addition of *Pseudomonas* *fluorescens* HYK0210‐SK09 to Mitigate Blooms of the Diatom *Stephanodiscus* *hantzschii* in Small‐ and Large‐Scale Mesocosms.” Journal of Applied Phycology 22: 409–419.

[emi470305-bib-0068] Kahla, O. , S. Melliti Ben Garali , F. Karray , et al. 2021. “Efficiency of Benthic Diatom‐Associated Bacteria in the Removal of Benzo(a)pyrene and Fluoranthene.” Science of the Total Environment 751: 141399. 32866829 10.1016/j.scitotenv.2020.141399

[emi470305-bib-0069] Karlson, B. , P. Andersen , L. Arneborg , et al. 2021. “Harmful Algal Blooms and Their Effects in Coastal Seas of Northern Europe.” Harmful Algae 102: 101989. 33875185 10.1016/j.hal.2021.101989

[emi470305-bib-0070] Kong, Y. , Z. Zhang , and Y. Peng . 2022. “Multi‐Objective Optimization of Ultrasonic Algae Removal Technology by Using Response Surface Method and Non‐Dominated Sorting Genetic Algorithm‐II.” Ecotoxicology and Environmental Safety 230: 113151. 34990992 10.1016/j.ecoenv.2021.113151

[emi470305-bib-0071] Lacal, J. , C. García‐Fontana , F. Muñoz‐Martínez , J. Ramos , and T. Krell . 2010. “Sensing of Environmental Signals: Classification of Chemoreceptors According to the Size of Their Ligand Binding Regions.” Environmental Microbiology 12: 2873–2884. 20738376 10.1111/j.1462-2920.2010.02325.x

[emi470305-bib-0072] Lan, J. , P. Liu , X. Hu , and S. Zhu . 2024. “Harmful Algal Blooms in Eutrophic Marine Environments: Causes, Monitoring, and Treatment.” Water 16: 2525.

[emi470305-bib-0073] Le, V. V. , S.‐R. Ko , M. Kang , S.‐A. Lee , H.‐M. Oh , and C.‐Y. Ahn . 2022. “Algicide Capacity of *Paucibacter* *aquatile* DH15 on *Microcystis* *aeruginosa* by Attachment and Non‐Attachment Effects.” Environmental Pollution 302: 119079. 35245623 10.1016/j.envpol.2022.119079

[emi470305-bib-0074] Lee, T. C.‐H. , W. Lam , N. F.‐Y. Tam , S. J.‐L. Xu , C.‐L. Lee , and F. W.‐F. Lee . 2024. “Proteomic Insights of Interaction Between Ichthyotoxic Dinoflagellate *Karenia* *mikimotoi* and Algicidal Bacteria *Maribacter* *dokdonensis* .” Marine Pollution Bulletin 209: 117227. 39500172 10.1016/j.marpolbul.2024.117227

[emi470305-bib-0075] Li, C. , S. Song , Y. Liu , and T. Chen . 2014. “Occurrence of Amoebophrya spp. Infection in Planktonic Dinoflagellates in Changjiang (Yangtze River) Estuary, China.” Harmful Algae 37: 117–124.

[emi470305-bib-0076] Li, D. , X. Chen , Y. Wang , et al. 2024. “Panoptic Elucidation of Algicidal Mechanism of *Raoultella* sp. S1 Against the *Microcystis* *aeruginosa* by TMT Quantitative Proteomics.” Chemosphere 352: 141287. 38272139 10.1016/j.chemosphere.2024.141287

[emi470305-bib-0077] Li, H. , R. Xing , X. Ji , et al. 2024. “Natural Algicidal Compounds: Strategies for Controlling Harmful Algae and Application.” Plant Physiology and Biochemistry 215: 108981. 39163650 10.1016/j.plaphy.2024.108981

[emi470305-bib-0078] Li, J. , F. Azam , and S. Zhang . 2016. “Outer Membrane Vesicles Containing Signalling Molecules and Active Hydrolytic Enzymes Released by a Coral Pathogen *Vibrio shilonii* AK1.” Environmental Microbiology 18: 3850–3866. 27102379 10.1111/1462-2920.13344

[emi470305-bib-0079] Li, J. , H. Gu , V. J. Lovko , et al. 2024. “The Ciliate Euplotes Balteatus Exhibits Removal Capacity Upon the Dinoflagellates Karenia Mikimotoi and Prorocentrum Shikokuense.” Harmful Algae 138: 102685. 39244228 10.1016/j.hal.2024.102685

[emi470305-bib-0080] Li, X. , T. Yan , J. Lin , R. Yu , and M. Zhou . 2017. “Detrimental Impacts of the Dinoflagellate Karenia Mikimotoi in Fujian Coastal Waters on Typical Marine Organisms.” Harmful Algae 61: 1–12.

[emi470305-bib-0081] Li, Y. , X. Lei , H. Zhu , et al. 2016. “Chitinase Producing Bacteria With Direct Algicidal Activity on Marine Diatoms.” Scientific Reports 6: 21984. 26902175 10.1038/srep21984 PMC4763246

[emi470305-bib-0082] Li, Y. , Y. Wang , X. Lin , et al. 2024. “Algicidal Bacteria‐Derived Membrane Vesicles as Shuttles Mediating Cross‐Kingdom Interactions Between Bacteria and Algae.” Science Advances 10: eadn4526. 39110793 10.1126/sciadv.adn4526 PMC11305373

[emi470305-bib-0083] Li, Y. , H. Zhu , C. Guan , et al. 2014. “Towards Molecular, Physiological, and Biochemical Understanding of Photosynthetic Inhibition and Oxidative Stress in the Toxic *Alexandrium tamarense* Induced by a Marine Bacterium.” Applied Microbiology and Biotechnology 98: 4637–4652. 24682476 10.1007/s00253-014-5578-x

[emi470305-bib-0084] Li, Y. , H. Zhu , X. Lei , et al. 2015. “The Death Mechanism of the Harmful Algal Bloom Species *Alexandrium* *tamarense* Induced by Algicidal Bacterium *deinococcus* sp. Y35.” Frontiers in Microbiology 6: 992. 10.3389/fmicb.2015.00992. 26441921 PMC4585090

[emi470305-bib-0085] Li, Z. , S. Lin , X. Liu , J. Tan , J. Pan , and H. Yang . 2014. “A Freshwater Bacterial Strain, *Shewanella* sp. Lzh‐2, Isolated From Lake Taihu and Its Two Algicidal Active Substances, Hexahydropyrrolo[1,2‐a]Pyrazine‐1,4‐Dione and 2, 3‐Indolinedione.” Applied Microbiology and Biotechnology 98: 4737–4748. 24566920 10.1007/s00253-014-5602-1

[emi470305-bib-0086] Lim, C. C. , J. Yoon , K. Reynolds , et al. 2023. “Harmful Algal Bloom Aerosols and Human Health.” eBioMedicine 93: 104604. 37164781 10.1016/j.ebiom.2023.104604 PMC10363441

[emi470305-bib-0087] Lin, Q. , J. Feng , Z. Hu , R. Cai , and H. Wang . 2023. “ROS‐Dependent Cell Death of *Heterosigma* *akashiwo* Induced by Algicidal Bacterium Hahella sp. KA22.” Marine Genomics 69: 101027. 36921441 10.1016/j.margen.2023.101027

[emi470305-bib-0088] Lin, S. 2024. “A Decade of Dinoflagellate Genomics Illuminating an Enigmatic Eukaryote Cell.” BMC Genomics 25: 932. 39367346 10.1186/s12864-024-10847-5 PMC11453091

[emi470305-bib-0089] Lin, T. , Y. Feng , W. Miao , et al. 2024. “Elevated Temperature Alters Bacterial Community From Mutualism to Antagonism With *Skeletonema* *costatum* : Insights Into the Role of a Novel Species, *Tamlana* sp. MS1.” mSphere 9: e00198‐24. 38940599 10.1128/msphere.00198-24 PMC11288006

[emi470305-bib-0090] Lipsman, V. , O. Shlakhter , J. Rocha , and E. Segev . 2024. “Bacteria Contribute Exopolysaccharides to an Algal‐Bacterial Joint Extracellular Matrix.” npj Biofilms and Microbiomes 10: 36. 38561371 10.1038/s41522-024-00510-y PMC10984933

[emi470305-bib-0091] Liu, C. , N. Ma , M. Sun , et al. 2025. “Impact of Climate Change on Frequency and Community Structure of Red Tide Events in the Northern South China Sea.” Climate Dynamics 63: 51.

[emi470305-bib-0092] Liu, F. 2023. “Applications‐Oriented Algicidal Efficacy Research and In‐Depth Mechanism of a Novel Strain *Brevibacillus* sp. on *Microcystis* *aeruginosa* .” Environmental Pollution 330: 121812. 37178955 10.1016/j.envpol.2023.121812

[emi470305-bib-0093] Liu, F. , S. Feng , A. Ali Nasser Mansoor Al‐Haimi , et al. 2024. “Discovery of Two Novel Bioactive Algicidal Substances From *Brevibacillus* sp. via Metabolomics Profiling and Back‐Validation.” Journal of Hazardous Materials 469: 133985. 38471378 10.1016/j.jhazmat.2024.133985

[emi470305-bib-0094] Liu, J. , K. Liu , Z. Zhao , et al. 2022. “The LuxS/AI‐2 Quorum‐Sensing System Regulates the Algicidal Activity of *Shewanella* *xiamenensis* Lzh‐2.” Frontiers in Microbiology 12: 814929. 35154040 10.3389/fmicb.2021.814929 PMC8831721

[emi470305-bib-0095] Liu, Z. Z. 2014. “Effects of Freshwater Bacterial Siderophore on Microcystis and Anabaena.” Biological Control 78: 42–48. 10.1016/j.biocontrol.2014.07.010.

[emi470305-bib-0096] Low, P. S. , and J. R. Merida . 1996. “The Oxidative Burst in Plant Defense: Function and Signal Transduction.” Physiologia Plantarum 96: 533–542.

[emi470305-bib-0097] Lu, X. , B. Zhou , L. Xu , et al. 2016. “A Marine Algicidal Thalassospira and Its Active Substance Against the Harmful Algal Bloom Species *Karenia* *mikimotoi* .” Applied Microbiology and Biotechnology 100: 5131–5139. 26846742 10.1007/s00253-016-7352-8

[emi470305-bib-0098] Mallick, N. , and F. H. Mohn . 2000. “Reactive Oxygen Species: Response of Algal Cells.” Journal of Plant Physiology 157: 183–193.

[emi470305-bib-0099] Martens‐Habbena, W. , P. M. Berube , H. Urakawa , J. R. De La Torre , and D. A. Stahl . 2009. “Ammonia Oxidation Kinetics Determine Niche Separation of Nitrifying Archaea and Bacteria.” Nature 461: 976–979. 19794413 10.1038/nature08465

[emi470305-bib-0100] Mayali, X. , P. Franks , and R. Burton . 2011. “Temporal Attachment Dynamics by Distinct Bacterial Taxa During a Dinoflagellate Bloom.” Aquatic Microbial Ecology 63: 111–122.

[emi470305-bib-0101] Mazur, H. , and E. Homme . 1993. “Presence of Auxin Indole‐3‐Acetic Acid in the Northern Adriatic Sea: Phytohormones and Mucilage.” Marine Ecology Progress Series 99: 163–168.

[emi470305-bib-0102] McBride, M. J. 2001. “Bacterial Gliding Motility: Multiple Mechanisms for Cell Movement Over Surfaces.” Annual Review of Microbiology 55: 49–75. 10.1146/annurev.micro.55.1.49 11544349

[emi470305-bib-0103] Meyer, N. , A. Bigalke , A. Kaulfuß , and G. Pohnert . 2017. “Strategies and Ecological Roles of Algicidal Bacteria.” FEMS Microbiology Reviews 41: 880–899. 28961821 10.1093/femsre/fux029

[emi470305-bib-0104] Miller, T. R. , and R. Belas . 2006. “Motility Is Involved in *Silicibacter* sp. TM1040 Interaction With Dinoflagellates.” Environmental Microbiology 8: 1648–1659. 16913924 10.1111/j.1462-2920.2006.01071.x

[emi470305-bib-0105] Miyamoto, K. , M. Okunishi , E. Nukui , et al. 2007. “The Regulator CdsS/CdsR Two‐Component System Modulates Expression of Genes Involved in Chitin Degradation of *Pseudoalteromonas piscicida* Strain O‐7.” Archives of Microbiology 188: 619–628. 17634925 10.1007/s00203-007-0283-0

[emi470305-bib-0106] Ni, L. , C. Zhu , C. Du , Y. Fang , J. Wang , and S. Li . 2023. “Characterization of a Novel Artemisinin Algicidal Particle and Its Inhibitory Effect on *Microcystis* *aeruginosa* .” Bulletin of Environmental Contamination and Toxicology 110: 82. 37086296 10.1007/s00128-023-03718-4

[emi470305-bib-0107] Noh, S. Y. , S. W. Jung , B. H. Kim , T. Katano , and M.‐S. Han . 2017. “Algicidal Activity of the Bacterium, *Pseudomonas* *fluorescens* SK09, to Mitigate *Stephanodiscus* *hantzschii* (Bacillariophyceae) Blooms Using Field Mesocosms.” Journal of Freshwater Ecology 32: 477–488.

[emi470305-bib-0108] O'Toole, G. , H. B. Kaplan , and R. Kolter . 2000. “Biofilm Formation as Microbial Development.” Annual Review of Microbiology 54: 49–79. 10.1146/annurev.micro.54.1.49 11018124

[emi470305-bib-0109] Paerl, H. W. , R. S. Fulton , P. H. Moisander , and J. Dyble . 2001. “Harmful Freshwater Algal Blooms, With an Emphasis on Cyanobacteria.” Scientific World Journal 1: 76–113. 10.1100/tsw.2001.16 PMC6083932 12805693

[emi470305-bib-0110] Paerl, H. W. , and J. Huisman . 2008. “Blooms Like It Hot.” Science 320: 57–58. 18388279 10.1126/science.1155398

[emi470305-bib-0111] Park, J. , I. Yoshinaga , T. Nishikawa , and I. Imai . 2010. “Algicidal Bacteria in Particle‐Associated Form and in Free‐Living Form During a Diatom Bloom in the Seto Inland Sea, Japan.” Aquatic Microbial Ecology 60: 151–161.

[emi470305-bib-0112] Park, K. S. , D. Choi , H. K. Son , Y.‐C. Chang , and H. Cho . 2023. “An Algicidal Agent Against Harmful Algae Using Novel N1‐Benzyl‐N3, N3‐Diethylpropane‐1,3‐Diamine Derivatives.” Biotechnology and Bioprocess Engineering 28: 215–225.

[emi470305-bib-0113] Parkinson, J. S. 2015. “Signaling and Sensory Adaptation in *Escherichia* *coli* Chemoreceptors: 2015 Update.” Trends in Microbiology 23: 257–266. 10.1016/j.tim.2015.03.003. 25834953 PMC4417406

[emi470305-bib-0114] Parveen, S. , and S. K. Patidar . 2023. “Bacterial Quorum Sensing Precursors N‐(3‐Oxododecanoyl)‐L‐Homoserine Lactone & N‐(3‐Hydroxyoctanoyl)‐DL‐Homoserine Lactone Ameliorate Algal Biomass, Lipids and Fatty Acids.” Chemical Engineering Journal 471: 144757.

[emi470305-bib-0115] Patidar, S. K. 2025. “Metabolic Interactions Between Microalgae and Bacteria: Multifunctional Ecological Interplay and Environmental Applications.” Algal Research 86: 103904.

[emi470305-bib-0116] Paul, C. , and G. Pohnert . 2011. “Interactions of the Algicidal Bacterium *Kordia algicida* With Diatoms: Regulated Protease Excretion for Specific Algal Lysis.” PLoS One 6: e21032. 21695044 10.1371/journal.pone.0021032 PMC3117869

[emi470305-bib-0117] Pérez‐Pérez, M. E. , S. D. Lemaire , and J. L. Crespo . 2012. “Reactive Oxygen Species and Autophagy in Plants and Algae.” Plant Physiology 160: 156–164. 22744983 10.1104/pp.112.199992 PMC3440194

[emi470305-bib-0118] Persat, A. , Y. F. Inclan , J. N. Engel , H. A. Stone , and Z. Gitai . 2015. “Type IV Pili Mechanochemically Regulate Virulence Factors in *Pseudomonas aeruginosa* .” Proceedings of the National Academy of Sciences of the United States of America 112: 7563–7568. 26041805 10.1073/pnas.1502025112 PMC4475988

[emi470305-bib-0119] Pospíšil, P. 2012. “Molecular Mechanisms of Production and Scavenging of Reactive Oxygen Species by Photosystem II.” Biochimica et Biophysica Acta (BBA) ‐ Bioenergetics 1817: 218–231. 21641332 10.1016/j.bbabio.2011.05.017

[emi470305-bib-0120] Prakash, P. , Y. Baig , F. J. Peaudecerf , and R. E. Goldstein . 2025. “Dynamics of an Algae–Bacteria Microcosm: Photosynthesis, Chemotaxis, and Expulsion in Inhomogeneous Active Matter.” Proceedings of the National Academy of Sciences of the United States of America 122: e2410225122. 40096603 10.1073/pnas.2410225122 PMC11962504

[emi470305-bib-0121] Qin, B. , G. Zhu , G. Gao , et al. 2010. “A Drinking Water Crisis in Lake Taihu, China: Linkage to Climatic Variability and Lake Management.” Environmental Management 45: 105–112. 19915899 10.1007/s00267-009-9393-6

[emi470305-bib-0122] Ray, S. , and S. N. Bagchi . 2001. “Nutrients and pH Regulate Algicide Accumulation in Cultures of the Cyanobacterium *Oscillatoria laetevirens* .” New Phytologist 149: 455–460. 33873328 10.1046/j.1469-8137.2001.00061.x

[emi470305-bib-0123] Reinfelder, J. R. 2011. “Carbon Concentrating Mechanisms in Eukaryotic Marine Phytoplankton.” Annual Review of Marine Science 3: 291–315. 10.1146/annurev-marine-120709-142720 21329207

[emi470305-bib-0124] Risgaard‐Petersen, N. , M. H. Nicolaisen , N. P. Revsbech , and B. A. Lomstein . 2004. “Competition Between Ammonia‐Oxidizing Bacteria and Benthic Microalgae.” Applied and Environmental Microbiology 70: 5528–5537. 15345441 10.1128/AEM.70.9.5528-5537.2004 PMC520845

[emi470305-bib-0125] Rose, M. M. , D. Scheer , Y. Hou , et al. 2021. “The Bacterium Pseudomonas Protegens Antagonizes the Microalga *Chlamydomonas* *reinhardtii* Using a Blend of Toxins.” Environmental Microbiology 23: 5525–5540. 10.1111/1462-2920.15700. 34347373

[emi470305-bib-0126] Roth, P. B. , M. J. Twiner , C. M. Mikulski , A. B. Barnhorst , and G. J. Doucette . 2008. “Comparative Analysis of Two Algicidal Bacteria Active Against the Red Tide Dinoflagellate Karenia *brevis* .” Harmful Algae 7: 682–691.

[emi470305-bib-0127] Sakamoto, S. , W. A. Lim , D. Lu , X. Dai , T. Orlova , and M. Iwataki . 2021. “Harmful Algal Blooms and Associated Fisheries Damage in East Asia: Current Status and Trends in China, Japan, Korea and Russia.” Harmful Algae 102: 101787. 33875176 10.1016/j.hal.2020.101787

[emi470305-bib-0128] Schatz, D. 2018. “Extracellular Vesicles — New Players in Cell–Cell Communication in Aquatic Environments.” Current Opinion in Microbiology 43: 148–154. 10.1016/j.mib.2018.01.014. 29448174

[emi470305-bib-0129] Schatz, D. , S. Rosenwasser , S. Malitsky , S. G. Wolf , E. Feldmesser , and A. Vardi . 2017. “Communication via Extracellular Vesicles Enhances Viral Infection of a Cosmopolitan Alga.” Nature Microbiology 2: 1485–1492. 10.1038/s41564-017-0024-3 28924189

[emi470305-bib-0130] Segev, E. , T. P. Wyche , K. H. Kim , et al. 2016. “Dynamic Metabolic Exchange Governs a Marine Algal‐Bacterial Interaction.” eLife 5: e17473. 27855786 10.7554/eLife.17473 PMC5148602

[emi470305-bib-0131] Seyedsayamdost, M. R. , R. J. Case , R. Kolter , and J. Clardy . 2011. “The Jekyll‐and‐Hyde Chemistry of *Phaeobacter* *gallaeciensis* .” Nature Chemistry 3: 331–335. 10.1038/nchem.1002 PMC3376411 21430694

[emi470305-bib-0132] Seymour, J. R. , T. Ahmed , Marcos , and R. Stocker . 2008. “A Microfluidic Chemotaxis Assay to Study Microbial Behavior in Diffusing Nutrient Patches.” Limnology and Oceanography: Methods 6: 477–488.

[emi470305-bib-0133] Seymour, J. R. , T. Ahmed , and R. Stocker . 2009. “Bacterial Chemotaxis Towards the Extracellular Products of the Toxic Phytoplankton *Heterosigma* *akashiwo* .” Journal of Plankton Research 31: 1557–1561.

[emi470305-bib-0134] Seymour, J. R. , S. A. Amin , J.‐B. Raina , and R. Stocker . 2017. “Zooming in on the Phycosphere: The Ecological Interface for Phytoplankton–Bacteria Relationships.” Nature Microbiology 2: 17065. 10.1038/nmicrobiol.2017.65. 28555622

[emi470305-bib-0135] Seymour, J. R. , R. Simó , T. Ahmed , and R. Stocker . 2010. “Chemoattraction to Dimethylsulfoniopropionate Throughout the Marine Microbial Food Web.” Science 329: 342–345. 20647471 10.1126/science.1188418

[emi470305-bib-0136] Shi, J. , W. Wang , F. Wang , et al. 2023. “Efficient Inactivation of Harmful Algae *K*. *mikimotoi* by a Novel Algicidal Bacterium via a Rare Direct Contact Pathway: Performances and Mechanisms.” Science of the Total Environment 892: 164401. 37247737 10.1016/j.scitotenv.2023.164401

[emi470305-bib-0137] Shi, R. , H. Huang , Z. Qi , W. Hu , Z. Tian , and M. Dai . 2013. “Algicidal Activity Against *Skeletonema costatum* by Marine Bacteria Isolated From a High Frequency Harmful Algal Blooms Area in Southern Chinese Coast.” World Journal of Microbiology and Biotechnology 29: 153–162. 23054696 10.1007/s11274-012-1168-1

[emi470305-bib-0138] Shi, X. , L. Liu , Y. Li , et al. 2018. “Isolation of an Algicidal Bacterium and Its Effects Against the Harmful‐Algalbloom Dinoflagellate Prorocentrum Donghaiense (Dinophyceae).” Harmful Algae 80: 72–79. 30502814 10.1016/j.hal.2018.09.003

[emi470305-bib-0139] Sjoblad, R. D. , and R. Mitchell . 1979. “Chemotactic Responses of *Vibrio alginolyticus* to Algal Extracellular Products.” Canadian Journal of Microbiology 25: 964–967. 396019 10.1139/m79-147

[emi470305-bib-0140] Slightom, R. N. , and A. Buchan . 2009. “Surface Colonization by Marine Roseobacters: Integrating Genotype and Phenotype.” Applied and Environmental Microbiology 75: 6027–6037. 19666726 10.1128/AEM.01508-09 PMC2753062

[emi470305-bib-0141] Smayda, T. J. 1997. “Harmful Algal Blooms: Their Ecophysiology and General Relevance to Phytoplankton Blooms in the Sea.” Limnology and Oceanography 42: 1137–1153.

[emi470305-bib-0142] Son, K. , F. Menolascina , and R. Stocker . 2016. “Speed‐Dependent Chemotactic Precision in Marine Bacteria.” Proceedings of the National Academy of Sciences 113: 8624–8629. 10.1073/pnas.1602307113 PMC4978249 27439872

[emi470305-bib-0143] Sonnenschein, E. C. , D. A. Syit , H.‐P. Grossart , and M. S. Ullrich . 2012. “Chemotaxis of Marinobacter Adhaerens and Its Impact on Attachment to the Diatom *Thalassiosira weissflogii* .” Applied and Environmental Microbiology 78: 6900–6907. 22820333 10.1128/AEM.01790-12 PMC3457506

[emi470305-bib-0144] Steele, D. J. , D. J. Franklin , and G. J. C. Underwood . 2014. “Protection of Cells From Salinity Stress by Extracellular Polymeric Substances in Diatom Biofilms.” Biofouling 30: 987–998. 25268215 10.1080/08927014.2014.960859 PMC4706044

[emi470305-bib-0145] Stock, F. , G. Bilcke , S. De Decker , et al. 2020. “Distinctive Growth and Transcriptional Changes of the Diatom Seminavis Robusta in Response to Quorum Sensing Related Compounds.” Frontiers in Microbiology 11: 1240. 32582129 10.3389/fmicb.2020.01240 PMC7296067

[emi470305-bib-0146] Stocker, R. , and J. R. Seymour . 2012. “Ecology and Physics of Bacterial Chemotaxis in the Ocean.” Microbiology and Molecular Biology Reviews 76: 792–812. 23204367 10.1128/MMBR.00029-12 PMC3510523

[emi470305-bib-0147] Stocker, R. , J. R. Seymour , A. Samadani , D. E. Hunt , and M. F. Polz . 2008. “Rapid Chemotactic Response Enables Marine Bacteria to Exploit Ephemeral Microscale Nutrient Patches.” Proceedings of the National Academy of Sciences 105: 4209–4214. 10.1073/pnas.0709765105 PMC2393791 18337491

[emi470305-bib-0148] Takano, Y. , Y. Tomaru , and K. Nagasaki . 2018. “Visualization of a Dinoflagellate‐Infecting Virus HcDNAV and Its Infection Process.” Viruses 10: 554. 30314306 10.3390/v10100554 PMC6212932

[emi470305-bib-0149] Tang, D. , X. Li , L. Zhang , et al. 2025. “Reactive Oxygen Species‐Mediated Signal Transduction and Utilization Strategies in Microalgae.” Bioresource Technology 418: 132004. 39710205 10.1016/j.biortech.2024.132004

[emi470305-bib-0150] Teplitski, M. , H. Chen , S. Rajamani , et al. 2004. “ *Chlamydomonas reinhardtii* Secretes Compounds That Mimic Bacterial Signals and Interfere With Quorum Sensing Regulation in Bacteria.” Plant Physiology 134: 137–146. 14671013 10.1104/pp.103.029918 PMC316294

[emi470305-bib-0151] Tsikas, D. 2017. “Assessment of Lipid Peroxidation by Measuring Malondialdehyde (MDA) and Relatives in Biological Samples: Analytical and Biological Challenges.” Analytical Biochemistry 524: 13–30. 27789233 10.1016/j.ab.2016.10.021

[emi470305-bib-0152] Van Tol, H. M. , S. A. Amin , and E. V. Armbrust . 2017. “Ubiquitous Marine Bacterium Inhibits Diatom Cell Division.” ISME Journal 11: 31–42. 27623332 10.1038/ismej.2016.112 PMC5315476

[emi470305-bib-0153] Vanelslander, B. , C. Paul , J. Grueneberg , et al. 2012. “Daily Bursts of Biogenic Cyanogen Bromide (BrCN) Control Biofilm Formation Around a Marine Benthic Diatom.” Proceedings of the National Academy of Sciences of the United States of America 109: 2412–2417. 22308324 10.1073/pnas.1108062109 PMC3289321

[emi470305-bib-0154] Wang, H. , A. F. Bouwman , J. Van Gils , et al. 2023. “Hindcasting Harmful Algal Bloom Risk due to Land‐Based Nutrient Pollution in the Eastern Chinese Coastal Seas.” Water Research 231: 119669. 36716567 10.1016/j.watres.2023.119669

[emi470305-bib-0155] Wang, J. , A. R. J. Curson , S. Zhou , et al. 2024. “Alternative Dimethylsulfoniopropionate Biosynthesis Enzymes in Diverse and Abundant Microorganisms.” Nature Microbiology 9: 1979–1992. 10.1038/s41564-024-01715-9. PMC11306096 38862603

[emi470305-bib-0156] Wang, R. , and M. R. Seyedsayamdost . 2017. “Roseochelin B, an Algaecidal Natural Product Synthesized by the *Roseobacter Phaeobacter inhibens * in Response to Algal Sinapic Acid.” Organic Letters 19: 5138–5141. 28920692 10.1021/acs.orglett.7b02424

[emi470305-bib-0157] Wang, W. , H. Shen , P. Shi , J. Chen , L. Ni , and P. Xie . 2016. “Experimental Evidence for the Role of Heterotrophic Bacteria in the Formation of Microcystis Colonies.” Journal of Applied Phycology 28: 1111–1123.

[emi470305-bib-0158] Wang, Y. , and K. J. Coyne . 2020. “Immobilization of Algicidal Bacterium Shewanella sp. IRI‐160 and Its Application to Control Harmful Dinoflagellates.” Harmful Algae 94: 101798. 32414500 10.1016/j.hal.2020.101798

[emi470305-bib-0159] Wang, Y. , and K. J. Coyne . 2023. “Transcriptome Profiling Reveals a Global Response in Harmful Dinoflagellate *Karlodinium* *veneficum* to Naturally‐Occurring Bacterial Algicides.” Frontiers in Marine Science 10: 1112913.

[emi470305-bib-0160] Wei, Y. , J. Chen , M. Wang , et al. 2024. “The Double Heterostructure Photocatalyst MoS2@ZIF‐67/TiO_2_: Triumphant Modification of TiO_2_ and Efficient Removal of Karenia Mikimotoi.” Chemical Engineering Journal 479: 147954.

[emi470305-bib-0161] Whalen, K. E. , C. Kirby , R. M. Nicholson , M. O'Reilly , B. S. Moore , and E. L. Harvey . 2018. “The Chemical Cue Tetrabromopyrrole Induces Rapid Cellular Stress and Mortality in Phytoplankton.” Scientific Reports 8: 15498. 30341338 10.1038/s41598-018-33945-3 PMC6195506

[emi470305-bib-0162] Williams, P. , K. Winzer , W. C. Chan , and M. Cámara . 2007. “Look Who's Talking: Communication and Quorum Sensing in the Bacterial World.” Philosophical Transactions of the Royal Society, B: Biological Sciences 362: 1119–1134. 10.1098/rstb.2007.2039 PMC2435577 17360280

[emi470305-bib-0163] Woyke, T. , G. Xie , A. Copeland , et al. 2009. “Assembling the Marine Metagenome, One Cell at a Time.” PLoS One 4: e5299. 19390573 10.1371/journal.pone.0005299 PMC2668756

[emi470305-bib-0164] Wu, L. , X. Guo , X. Liu , and H. Yang . 2017. “NprR‐NprX Quorum‐Sensing System Regulates the Algicidal Activity of *Bacillus* sp. Strain S51107 Against Bloom‐Forming Cyanobacterium *Microcystis* *aeruginosa* .” Frontiers in Microbiology 8: 1968. 29075240 10.3389/fmicb.2017.01968 PMC5641580

[emi470305-bib-0165] Wurtsbaugh, W. A. , H. W. Paerl , and W. K. Dodds . 2019. “Nutrients, Eutrophication and Harmful Algal Blooms Along the Freshwater to Marine Continuum.” WIREs Water 6: e1373. 10.1002/wat2.1373.

[emi470305-bib-0166] Xie, Y. , H. Zhang , B. Cui , et al. 2024. “Enhanced Inhibitory Efficiency Against Toxic Bloom Forming *Raphidiopsis raciborskii * by *Streptomyces* sp. HY Through Triple Algicidal Modes: Direct and Indirect Attacks Combined With Bioflocculation.” Journal of Hazardous Materials 477: 135152. 39047554 10.1016/j.jhazmat.2024.135152

[emi470305-bib-0167] Xu, M. , Y. Chen , L. Chen , et al. 2024. “Investigating the Molecular Mechanisms of *Pseudalteromonas* sp. LD‐B1's Algicidal Effects on the Harmful Alga Heterosigma Akashiwo.” Ecotoxicology and Environmental Safety 282: 116690. 38981394 10.1016/j.ecoenv.2024.116690

[emi470305-bib-0168] Xu, Q. , S. Ali , M. Afzal , et al. 2024. “Advancements in Bacterial Chemotaxis: Utilizing the Navigational Intelligence of Bacteria and Its Practical Applications.” Science of the Total Environment 931: 172967. 38705297 10.1016/j.scitotenv.2024.172967

[emi470305-bib-0169] Yang, I. , U. John , S. Beszteri , G. Glöckner , B. Krock , and A. Goesmann . 2010. “Comparative Gene Expression in Toxic Versus Non‐Toxic Strains of the Marine Dinoflagellate *Alexandrium* *minutum* .” BMC Genomics 11: 248. 20403159 10.1186/1471-2164-11-248 PMC2874808

[emi470305-bib-0170] Yang, Q. , G. S. J. Pande , Z. Wang , et al. 2017. “Indole Signalling and (Micro)algal Auxins Decrease the Virulence of *Vibrio* *campbellii* , a Major Pathogen of Aquatic Organisms.” Environmental Microbiology 19: 1987–2004. 28251783 10.1111/1462-2920.13714

[emi470305-bib-0171] Yang, S. , S. J. Williams , M. Courtney , and L. Burchill . 2025. “Warfare Under the Waves: A Review of Bacteria‐Derived Algaecidal Natural Products.” Natural Product Reports 42: 10.1039.D4NP00038B. 10.1039/d4np00038b 39749862

[emi470305-bib-0172] Yang, X. , Z. Liu , Y. Zhang , X. Shi , and Z. Wu . 2024. “Dinoflagellate–Bacteria Interactions: Physiology, Ecology, and Evolution.” Biology 13: 579. 39194517 10.3390/biology13080579 PMC11351557

[emi470305-bib-0173] Yu, J. , W. Xu , J. Wang , et al. 2024. “Contact‐Mediated Algicidal Mechanism of Vibrio Coralliirubri ACE001 Against the Harmful Alga Karenia Mikimotoi.” iScience 27: 111254. 39569365 10.1016/j.isci.2024.111254 PMC11576403

[emi470305-bib-0174] Yu, L. , T. Li , H. Li , M. Ma , L. Li , and S. Lin . 2023. “ *In Situ* Molecular Ecological Analyses Illuminate Distinct Factors Regulating Formation and Demise of a Harmful Dinoflagellate Bloom.” Microbiology Spectrum 11: e05157‐22. 37074171 10.1128/spectrum.05157-22 PMC10269597

[emi470305-bib-0175] Zeng, Y. , J. Wang , C. Yang , et al. 2021. “A *Streptomyces* *globisporus* Strain Kills *Microcystis* *aeruginosa* via Cell‐To‐Cell Contact.” Science of the Total Environment 769: 144489. 33465632 10.1016/j.scitotenv.2020.144489

[emi470305-bib-0176] Zhang, F. , Y. Fan , D. Zhang , et al. 2020. “Effect and Mechanism of the Algicidal Bacterium *Sulfitobacter* *porphyrae* ZFX1 on the Mitigation of Harmful Algal Blooms Caused by *Prorocentrum donghaiense* .” Environmental Pollution 263: 114475. 33618477 10.1016/j.envpol.2020.114475

[emi470305-bib-0177] Zhang, H. , J. Lv , Y. Peng , et al. 2014. “Cell Death in a Harmful Algal Bloom Causing Species *Alexandrium tamarense* Upon an Algicidal Bacterium Induction.” Applied Microbiology and Biotechnology 98: 7949–7958. 24962118 10.1007/s00253-014-5886-1

[emi470305-bib-0178] Zhang, Y. , J. Li , Z. Hu , et al. 2022. “Transcriptome Analysis Reveals the Algicidal Mechanism of *Brevibacillus* *laterosporus* Against *Microcystis* *aeruginosa* Through Multiple Metabolic Pathways.” Toxins 14: 492. 35878230 10.3390/toxins14070492 PMC9320710

[emi470305-bib-0179] Zhang, Y. , X. Wang , and Y. Sun . 2024. “A Newly Identified Algicidal Bacterium of *Pseudomonas* *fragi* YB2: Algicidal Compounds and Effects.” Journal of Hazardous Materials 478: 135490. 39141946 10.1016/j.jhazmat.2024.135490

[emi470305-bib-0180] Zhang, Z. , D. Li , R. Xie , et al. 2023. “Plastoquinone Synthesis Inhibition by Tetrabromo Biphenyldiol as a Widespread Algicidal Mechanism of Marine Bacteria.” ISME Journal 17: 1979–1992. 37679430 10.1038/s41396-023-01510-0 PMC10579414

[emi470305-bib-0181] Zhang, Z. , S. Nair , L. Tang , et al. 2021. “Long‐Term Survival of *Synechococcus* and Heterotrophic Bacteria Without External Nutrient Supply After Changes in Their Relationship From Antagonism to Mutualism.” MBio 12: e01614‐21. 34465027 10.1128/mBio.01614-21 PMC8406228

[emi470305-bib-0182] Zheng, L. , H. Lin , B. Balaji‐Prasath , et al. 2023. “A Novel Algicidal Properties of Fermentation Products From *Pseudomonas* sp. Ps3 Strain on the Toxic Red Tide Dinoflagellate Species.” Frontiers in Microbiology 14: 1146325. 37138597 10.3389/fmicb.2023.1146325 PMC10150927

[emi470305-bib-0183] Zhou, S. , Y. Shao , N. Gao , et al. 2014. “Effect of Chlorine Dioxide on Cyanobacterial Cell Integrity, Toxin Degradation and Disinfection By‐Product Formation.” Science of the Total Environment 482, no. 483: 208–213. 24651056 10.1016/j.scitotenv.2014.03.007

[emi470305-bib-0184] Zhou, Z. , J. Cao , M. Wu , et al. 2025. “ *Bacillus* sp. Enhances the Interspecific Competitiveness of Its Host *Cyclotella* *atomus* .” Aquaculture 595: 741577.

[emi470305-bib-0185] Zhu, X. , S. Chen , G. Luo , et al. 2022. “A Novel Algicidal Bacterium, *Microbulbifer* sp. YX04, Triggered Oxidative Damage and Autophagic Cell Death in *Phaeocystis* *globosa* , Which Causes Harmful Algal Blooms.” Microbiology Spectrum 10: e00934‐21. 35019679 10.1128/spectrum.00934-21 PMC8754136

[emi470305-bib-0186] Zingone, A. , L. Escalera , K. Aligizaki , et al. 2021. “Toxic Marine Microalgae and Noxious Blooms in the Mediterranean Sea: A Contribution to the Global HAB Status Report.” Harmful Algae 102: 101843. 33875177 10.1016/j.hal.2020.101843

